# Synthesis, Antitumor Activity Evaluation and Mechanistic Study of Novel Bis‐Heterocyclic Chalcones Against Liver Cancer

**DOI:** 10.1002/jcla.70154

**Published:** 2026-02-09

**Authors:** Zhifen Li, Jingbo Ma, Xiannian Lv, Lei Zhang, Hai Xie

**Affiliations:** ^1^ School of Chemistry and Chemical Engineering Shanxi Datong University Datong Shanxi P. R. China; ^2^ Department of Geriatrics Shenzhen People's Hospital (The Second Clinical Medical College, Jinan University, the First Affiliated Hospital, Southern University of Science and Technology) Shenzhen Guangdong P. R. China; ^3^ Department of Geriatrics Fifth People's Hospital of Datong City Datong Shanxi P. R. China

**Keywords:** cancer, chalcone, hepatocellular carcinoma, heterocyclic

## Abstract

**Background:**

Chalcones and heterocyclic compounds exhibit remarkably high activity in medicinal chemistry. In recent years, bis‐chalcones have been reported to possess excellent anticancer activity. We synthesized a series of bis‐heterocyclic chalcones via asymmetric chain synthesis, with the aim of making new discoveries in anticancer activity.

**Methods:**

Bis‐heterocyclic chalcones were synthesized via Claisen‐Schmidt condensation and alkylation reactions. The inhibitory activities of the synthesized compounds against Huh‐1, Huh‐7, and HepG2 cell lines were evaluated using the CCK‐8 assay. Furthermore, the mechanism of action of these compounds was explored through live/dead cell staining, flow cytometric analysis, and Western blotting experiments.

**Results:**

Twelve bis‐heterocyclic chalcone compounds were synthesized. All synthesized compounds were fully characterized by spectroscopic methods and evaluated for their cytotoxic activities against Huh‐1, Huh‐7, and HepG2 cell lines using the CCK‐8 assay at concentrations ranging from 0 to 100 μM. Among them, derivative **3f** exhibited the most potent cytotoxicity against Huh‐7 (IC_50_ = 8.40 μM) and Huh‐1 (IC_50_ = 6.75 μM), whereas HepG2 cells were most sensitive to compound **3d** (IC_50_ = 27.99 μM). The mechanisms underlying the antitumor effects of **3d** and **3f** were further investigated through live/dead cell staining, flow cytometry, and western blot analysis. The results demonstrate that both compounds effectively induce apoptosis in liver cancer cells.

**Conclusions:**

Bis‐heterocyclic chalcone compounds exhibit favorable inhibitory activity against hepatocellular carcinoma cells by inducing cell apoptosis, and thus can serve as a class of pharmaceutically active structural units for further in‐depth research and screening.

## Graphical abstract

Western blotting was performed to analyze the expression of Caspase 3 and Cleaved‐Caspase 3 in Huh1 cells following different treatments, which supports the finding that bis‐heterocyclic chalcones 3d and 3f can inhibit the activity of hepatocellular carcinoma cells by inducing apoptosis.

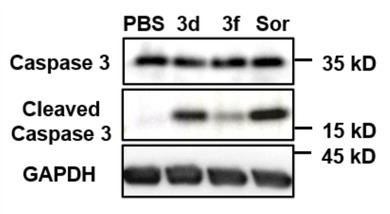

## 1 Introduction

Cancer remains one of the leading causes of death globally, with over 19.3 million new cases and approximately 10 million deaths reported in 2020 alone [1]. Specifically, liver cancer ranks as the fourth most common cause of cancer‐related deaths worldwide, and it is projected that nearly one million people will succumb to this disease by 2030 [2]. As highlighted above, the persistent annual rise in both cancer incidence and mortality has underscored the urgent need for developing highly effective pharmaceuticals to combat various types of cancers [3, 4, 5, 6, 7].

**FIGURE 1:**
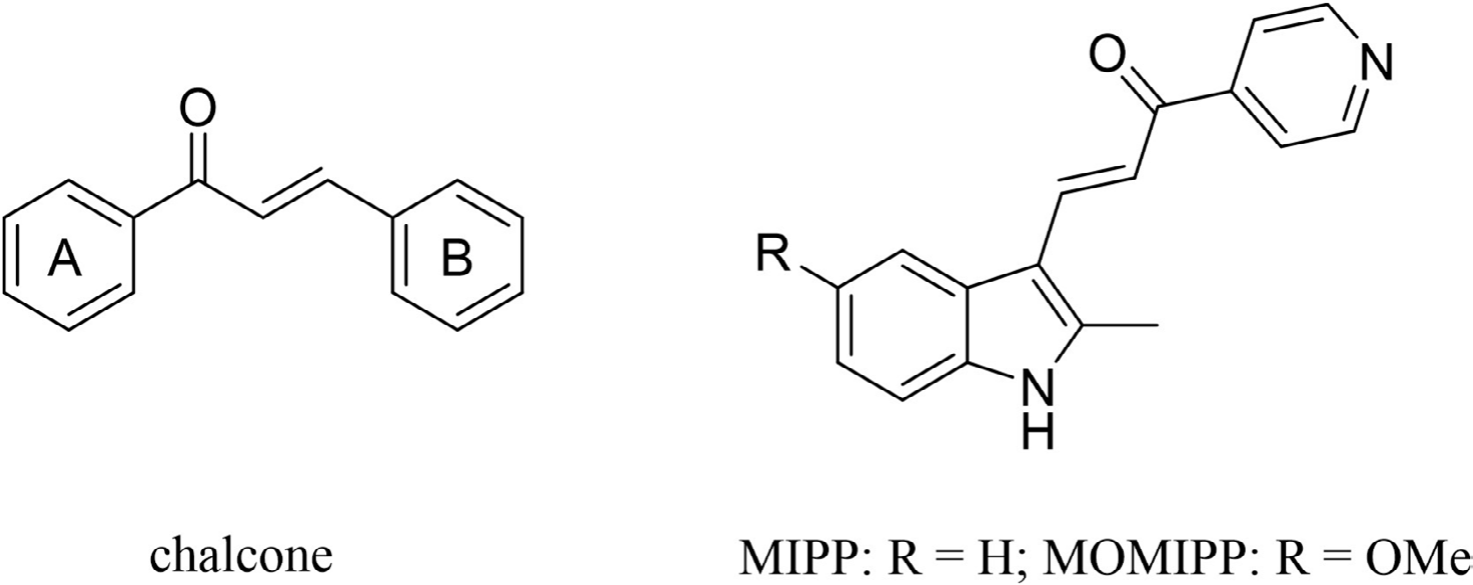
Structure of chalcone, MIPP and MOMIPP.

Heterocycles and chalcone derivatives (1,3‐diaryl‐2‐propen‐1‐ones) (Figure 1) exhibit diverse biological activities, including antibacterial, anti‐inflammatory, antimalarial, and antitubercular properties, thus occupying a pivotal role in new drug development [8, 9, 10, 11, 12, 13, 14, 15]. Notably, both heterocycles and chalcones have demonstrated significant anticancer activity in vitro and in vivo, highlighting their potential for the prevention and inhibition of various cancers [16, 17, 18, 19]. Consequently, the incorporation of heterocyclic moieties into chalcone structures may yield novel and more potent anticancer candidates [20]. For instance, heterocyclic chalcones such as MIPP and MOMIPP (indolyl‐pyridyl chalcones) (Figure 1) can induce methuosis—a unique form of programmed cell death distinct from apoptosis [21]. Additionally, dimeric compounds often exhibit enhanced biological activity compared to their corresponding monomeric counterparts [22, 23]. Our previous findings have shown that bis‐chalcone derivatives possess promising anticancer potential, making bis‐chalcone dimers a rational choice for the development of new anticancer agents [24].

Therefore, in the present study, 12 bis‐chalcones bearing heteroaryl moieties (furan, pyridine, and thiophene) were synthesized, and their cytotoxic activity against Huh‐1, Huh‐7, and HepG2 cell lines was evaluated using the CCK‐8 assay. The results indicate that certain bis‐chalcone derivatives exhibit potential as promising tumor inhibitory agents for specific hepatocellular carcinomas.

## 2 Materials and Methods

### 2.1 Chemistry

All materials were obtained from commercial suppliers. ^1^H NMR and ^13^C NMR spectra were recorded on a Varian INOVA spectrometer at 500 MHz and 126 MHz, respectively. Coupling constants (J) are reported in hertz (Hz), and multiplicities are denoted as s (singlet), d (doublet), t (triplet), q (quadruplet), and m (multiplet). High‐resolution mass spectrometry (HRMS) spectra were acquired using an Agilent 7250 and JEOL‐JMS‐T100LP AccuTOF Spectrometer.

Methyl N^2^, N^6^‐bis(2‐bromoacetyl) lysinate **1** and chalcones **2a‐l** were synthesized according to literature procedures [25, 26].

Synthesis of compounds **3a**‐**l** (General procedure). To a solution of methyl N^2^, N^6^‐bis(2‐bromoacetyl) lysinate (2 mmol) in DMF (8–10 mL) were added chalcones **2a‐l** (4 mmol) and K_2_CO_3_ (6 mmol). The reaction mixture was stirred at 60°C for 2 h, with reaction progress monitored by TLC. Upon complete consumption of the starting materials, the reaction mixture was poured into water (60 mL) and extracted with ethyl acetate (30 mL × 3). The combined organic layers were washed with brine (30 mL), dried over anhydrous sodium sulfate, and concentrated in vacuo. The crude products (**3b**, **3c**, **3e‐l**) were recrystallized from ethyl acetate to afford the pure products. Compounds **3a** and **3d** were purified by silica gel column chromatography (ethyl acetate/petroleum ether, 1:1) to yield the pure compounds as yellow or brown oils.

Methyl N^2^, N^6^‐bis(2‐(4‐((E)‐3‐(furan‐2‐yl)acryloyl)phenoxy)acetyl)lysinate (**3a**). Yellow oil, yield: 68%. ^1^H NMR (500 MHz, Chloroform‐*d*) δ 8.10–8.00 (m, 4H), 7.59 (d, *J* = 2.0 Hz, 1H), 7.56 (d, *J* = 1.9 Hz, 1H), 7.52 (d, *J* = 1.8 Hz, 2H), 7.45 (s, 1H), 7.42 (s, 1H), 7.08 (d, *J* = 8.2 Hz, 1H), 7.05–6.97 (m, 4H), 6.70 (t, *J* = 4.0 Hz, 2H), 6.67 (t, *J* = 5.9 Hz, 1H), 6.54–6.48 (m, 2H), 4.73–4.67 (m, 1H), 4.59 (d, *J* = 2.1 Hz, 2H), 4.53 (s, 2H), 3.75 (s, 3H), 3.38–3.26 (m, 2H), 1.97–1.88 (m, 1H), 1.81–1.71 (m, 1H), 1.63–1.51 (m, 2H), 1.40–1.29 (m, 2H) (Figures S1). ^13^C NMR (126 MHz, Chloroform‐*d*) δ 188.04, 187.97, 172.27, 167.59, 167.48, 160.73, 160.70, 151.74, 144.95, 132.49, 132.41, 130.92, 130.89, 130.48, 130.44, 118.95, 116.23, 116.20, 114.71, 114.59, 112.74, 67.32, 67.26, 52.63, 51.60, 38.69, 31.94, 28.99, 22.52 (Figure S2). HRMS (ESIMS) calculated for C_37_H_37_N_2_O_10_ [M + H]^+^: m/z 669.24427; found: 669.24394 (Figure S3).

Methyl N^2^, N^6^‐bis(2‐(4‐((E)‐3‐(5‐chlorofuran‐2‐yl)acryloyl)phenoxy)acetyl)lysinate **(3b)**. Light yellow solid, yield: 65%. ^1^H NMR (500 MHz, Chloroform‐*d*) δ 8.11–7.98 (m, 4H), 7.52–7.37 (m, 4H), 7.08 (d, *J* = 7.8 Hz, 1H), 7.06–6.95 (m, 4H), 6.71–6.62 (m, 3H), 6.33–6.27 (m, 2H), 4.74–4.67 (m, 1H), 4.59 (d, *J* = 2.0 Hz, 2H), 4.53 (s, 2H), 3.76 (s, 3H), 3.39–3.28 (m, 2H), 1.99–1.88 (m, 1H), 1.83–1.72 (m, 1H), 1.64–1.52 (m, 2H), 1.42–1.27 (m, 2H) (Figure S4). ^13^C NMR (126 MHz, Chloroform‐*d*) δ 187.62, 187.54, 172.26, 167.53, 167.43, 160.82, 160.78, 151.31, 139.55, 132.29, 132.21, 130.96, 130.93, 129.25, 129.22, 119.02, 117.97, 117.96, 114.73, 114.61, 109.62, 67.32, 67.26, 52.63, 51.59, 38.70, 31.93, 28.99, 22.52 (Figure S5). HRMS (ESIMS) calculated for C_37_H_35_N_2_O_10_Cl_2_ [M + H]^+^: m/z 737.16633; found: 737.16678 (Figure S6).

Methyl N^2^, N^6^‐bis(2‐(4‐((E)‐3‐(5‐bromofuran‐2‐yl)acryloyl)phenoxy)acetyl)lysinate **(3c)**. Yellow solid, yield: 70%. ^1^H NMR (500 MHz, Chloroform‐*d*) δ 8.10–8.00 (m, 4H), 7.51–7.39 (m, 4H), 7.09–6.95 (m, 5H), 6.64 (t, *J* = 3.8 Hz, 3H), 6.45 (dd, *J* = 3.5, 1.5 Hz, 2H), 4.73–4.67 (m, 1H), 4.59 (d, *J* = 2.0 Hz, 2H), 4.54 (s, 2H), 3.76 (s, 3H), 3.38–3.28 (m, 2H), 1.98–1.88 (m, 1H), 1.81–1.72 (m, 1H), 1.64–1.51 (m, 2H), 1.39–1.29 (m, 2H) (Figure S7). ^13^C NMR (126 MHz, Chloroform‐*d*) δ 187.75, 187.68, 172.31, 167.69, 167.53, 160.87, 160.85, 153.72, 132.42, 132.35, 131.06, 131.02, 129.22, 129.19, 125.85, 125.83, 119.31, 118.20, 118.18, 114.81, 114.70, 67.39, 67.34, 52.70, 51.66, 38.79, 32.04, 29.03, 22.58 (Figure S8). HRMS (ESIMS) calculated for C_37_H_35_N_2_O_10_Br_2_ [M + H]^+^: m/z 825.06530; found: 825.06561 (Figure S9).

Methyl N^2^, N^6^‐bis(2‐(4‐((E)‐3‐(5‐methylfuran‐2‐yl)acryloyl)phenoxy)acetyl)lysinate **(3d)**. Yellow solid, yield: 74%. ^1^H NMR (500 MHz, DMSO‐*d*_*6*_) δ 8.53 (d, *J* = 7.7 Hz, 1H), 8.21–8.00 (m, 5H), 7.55–7.37 (m, 4H), 7.07 (d, *J* = 8.5 Hz, 4H), 6.97 (d, *J* = 3.4 Hz, 2H), 6.32 (d, *J* = 3.4 Hz, 2H), 4.74–4.64 (m, 2H), 4.58 (s, 2H), 4.35–4.27 (m, 1H), 3.63 (s, 3H), 3.16–3.07(m, 2H), 2.37 (s, 6H), 1.81–1.65 (m, 2H), 1.49–1.38 (m, 2H), 1.33–1.25 (m, 2H) (Figure S10). ^13^C NMR (126 MHz, DMSO‐*d*_*6*_) δ 186.75, 172.32, 167.53, 166.99, 161.53, 161.48, 155.79, 149.96, 131.06, 130.46, 130.43, 129.88, 118.59, 116.92, 114.77, 109.75, 67.00, 66.60, 51.93, 51.67, 38.04, 30.28, 28.57, 22.72, 13.71 (Figure S11). HRMS (ESIMS) calculated for C_39_H_41_N_2_O_10_ [M + H]^+^: m/z 697.27557; found: 697.27546 (Figure S12).

Methyl N^2^, N^6^‐bis(2‐(4‐((E)‐3‐(thiophen‐2‐yl)acryloyl)phenoxy)acetyl)lysinate **(3e)**. Yellow solid, yield: 55%. ^1^H NMR (500 MHz, Chloroform‐*d*) δ 8.07–7.99 (m, 4H), 7.94 (s, 1H), 7.91 (s, 1H), 7.41 (d, *J* = 5.0, 2H), 7.36–7.31 (m, 3H), 7.30 (s, 1H), 7.14 (d, *J* = 8.2 Hz, 1H), 7.10–7.06 (m, 2H), 7.05–6.96 (m, 4H), 6.75(t, *J* = 6.2 Hz, 1H), 4.75–4.66 (m, 1H), 4.59 (d, *J* = 2.4 Hz, 2H), 4.52 (s, 2H), 3.75 (s, 3H), 3.39–3.26 (m, 2H), 1.98–1.88 (m, 1H), 1.82–1.73 (m, 1H), 1.64–1.52 (m, 2H), 1.41–1.29 (m, 2H) (Figure S13). ^13^C NMR (126 MHz, Chloroform‐*d*) δ 187.82, 187.74, 172.17, 167.41, 167.35, 160.62, 160.59, 140.27, 140.25, 136.79, 136.75, 132.15, 132.06, 132.02, 130.77, 130.74, 128.77, 128.75, 128.32, 120.13, 120.11, 114.57, 114.45, 67.15, 67.09, 52.52, 51.48, 38.54, 31.71, 28.87, 22.42 (Figure S14). HRMS (ESIMS) calculated for C_37_H_36_N_2_O_8_NaS_2_ [M + Na]^+^: m/z 723.18053; found: 723.18047 (Figure S15).

Methyl N^2^, N^6^‐bis(2‐(4‐((E)‐3‐(thiophen‐3‐yl)acryloyl)phenoxy)acetyl)lysinate **(3f)**. Yellow solid, yield: 57%. ^1^H NMR (500 MHz, Chloroform‐*d*) δ 8.03 (dd, *J* = 8.9, 3.2 Hz, 4H), 7.80 (d, *J* = 1.8 Hz, 1H), 7.77 (d, *J* = 1.8 Hz, 1H), 7.61–7.57 (m, 2H), 7.42 (d, *J* = 6.3 Hz, 2H), 7.39–7.35 (m, 3H), 7.33 (s, 1H), 7.06 (d, *J* = 8.2 Hz, 1H), 7.00 (dd, *J* = 16.5, 8.8 Hz, 4H), 6.65 (t, *J* = 6.0 Hz, 1H), 4.73–4.67 (m, 1H), 4.58 (d, *J* = 1.7 Hz, 2H), 4.52 (s, 2H), 3.75 (s, 3H), 3.38–3.24 (m, 2H), 1.97–1.88 (m, 1H), 1.81–1.70 (m, 1H), 1.63–1.51 (m, 2H), 1.39–1.26 (m, 2H) (Figure S16). ^13^C NMR (126 MHz, Chloroform‐*d*) δ 188.92, 188.84, 172.33, 167.55, 167.45, 160.66, 160.63, 138.25, 138.07, 138.03, 132.56, 132.48, 130.98, 130.95, 129.30, 129.28, 127.15, 125.33, 125.31, 121.32, 121.30, 114.70, 114.58, 67.31, 67.25, 52.73, 51.56, 38.68, 31.97, 29.00, 22.49 (Figure S17). HRMS (ESIMS) calculated for C_37_H_37_N_2_O_8_S_2_ [M + H]^+^: m/z 701.19858; found: 701.19946 (Figure S18).

Methyl N^2^, N^6^‐bis(2‐(4‐((E)‐3‐(5‐chlorothiophen‐2‐yl)acryloyl)phenoxy)acetyl)lysinate **(3g)**. Yellow solid, yield: 58%. ^1^H NMR (500 MHz, Chloroform‐*d*) δ 8.01 (dd, *J* = 8.9, 2.9 Hz, 4H), 7.80 (d, *J* = 2.4 Hz, 1H), 7.77 (d, *J* = 2.5 Hz, 1H), 7.20 (s, 1H), 7.17 (s, 1H), 7.12 (t, *J* = 4.5 Hz, 2H), 7.07 (d, *J* = 8.8 Hz 1H), 7.01 (dd, *J* = 15.7, 8.9 Hz, 4H), 6.91 (dd, *J* = 3.9, 2.1 Hz, 2H), 6.65 (t, *J* = 6.1 Hz, 1H), 4.74–4.67 (m, 1H), 4.59 (d, *J* = 1.3 Hz, 2H), 4.53 (s, 2H), 3.76 (s, 3H), 3.40–3.27 (m, 2H), 1.98–1.89 (m, 1H), 1.82–1.73 (m, 1H), 1.64–1.54 (m, 2H), 1.39–1.28 (m, 2H) (Figure S19). ^13^C NMR (126 MHz, Chloroform‐*d*) δ 187.66, 187.57, 172.33, 167.49, 167.40, 160.80, 160.76, 139.19, 136.34, 136.31, 133.60, 133.58, 132.22, 132.15, 131.78, 130.95, 130.92, 127.74, 120.22, 120.20, 114.75, 114.64, 67.31, 67.24, 52.72, 51.57, 38.69, 31.97, 29.00, 22.50 (Figure S20). HRMS (ESIMS) calculated for C_37_H_35_N_2_O_8_S_2_ Cl_2_[M + H]^+^: m/z 769.12064; found: 769.12151 (Figure S21).

Methyl N^2^, N^6^‐bis(2‐(4‐((E)‐3‐(5‐bromothiophen‐2‐yl)acryloyl)phenoxy)acetyl)lysinate **(3h)**. Yellow solid, yield: 50%. ^1^H NMR (500 MHz, Chloroform‐*d*) δ 8.01 (dd, *J* = 8.8, 3.5 Hz, 4H), 7.81 (d, *J* = 2.3 Hz, 1H), 7.78 (d, *J* = 2.3 Hz, 1H), 7.22 (s, 1H), 7.19 (s, 1H), 7.09 (t, *J* = 4.4 Hz, 3H), 7.06–7.03 (m, 3H), 7.03–6.97 (m, 3H), 6.66 (t, *J* = 6.0 Hz, 1H), 4.74–4.67 (m, 1H), 4.59 (s, 2H), 4.53 (s, 2H), 3.76 (s, 3H), 3.38–3.29 (m, 2H), 1.99–1.89 (m, 1H), 1.82–1.72 (m, 1H), 1.65–1.52 (m, 2H), 1.41–1.28 (m, 2H) (Figure S22). ^13^C NMR (126 MHz, Chloroform‐*d*) δ 187.65, 187.57, 172.31, 167.48, 167.38, 160.79, 160.76, 141.99, 136.03, 136.00, 132.43, 132.19, 132.12, 131.42, 130.94, 130.90, 120.49, 120.46, 116.39, 116.37, 114.74, 114.62, 67.29, 67.23, 52.71, 51.56, 38.68, 31.94, 28.99, 22.49 (Figure S23). HRMS (ESIMS) calculated for C_37_H_35_N_2_O_8_S_2_ Br_2_[M + H]^+^: m/z 857.01961; found: 857.02026 (Figure S24).

Methyl N^2^, N^6^‐bis(2‐(4‐((E)‐3‐(5‐methylthiophen‐2‐yl)acryloyl)phenoxy)acetyl)lysinate **(3i)**. Yellow solid, yield:55%. ^1^H NMR (500 MHz, Chloroform‐*d*) δ 8.04–7.97 (m, 4H), 7.88–7.79 (m, 2H), 7.19 (d, *J* = 1.7 Hz, 1H), 7.17–7.12 (m, 3H), 7.08 (d, *J* = 8.2, 1H), 7.05–6.95 (m, 4H), 6.76–6.71 (m, 2H), 6.71–6.65 (m, 1H), 4.72–4.66 (m, 1H), 4.58 (d, *J* = 1.9 Hz, 2H), 4.52 (s, 2H), 3.75 (d, *J* = 1.6 Hz, 3H), 3.38–3.26 (m, 2H), 2.51 (s, 6H), 1.96–1.89 (m, 1H), 1.81–1.70 (m, 1H), 1.63–1.53 (m, 2H), 1.39–1.28 (m, 2H) (Figure S25). ^13^C NMR (126 MHz, Chloroform‐*d*) δ 187.96, 187.88, 172.18, 167.51, 167.42, 160.57, 160.53, 144.50, 144.47, 138.39, 138.37, 137.30, 137.25, 132.87, 132.85, 132.45, 132.36, 130.71, 130.68, 126.86, 118.93, 114.58, 114.46, 67.24, 67.18, 52.51, 51.53, 38.60, 31.78, 28.90, 22.46, 15.86 (Figure S26). HRMS (ESIMS) calculated for C_39_H_41_N_2_O_8_S_2_ [M + H]^+^: m/z 729.22988; found: 729.22962 (Figure S27).

Methyl N^2^, N^6^‐bis(2‐(4‐((E)‐3‐(6‐chloropyridin‐3‐yl)acryloyl)phenoxy)acetyl)lysinate **(3j)**. White solid, yield: 74%. ^1^H NMR (500 MHz, DMSO‐*d*_6_) δ 8.86 (d, *J* = 2.5 Hz, 2H), 8.57 (d, *J* = 7.7 Hz, 1H), 8.48–8.39 (m, 2H), 8.25–8.15 (m, 5H), 8.12 (d, *J* = 4.3 Hz, 1H), 8.11 (d, *J* = 4.3 Hz, 1H), 7.72 (d, *J* = 15.7 Hz, 2H), 7.63 (d, *J* = 8.4 Hz, 2H), 7.10 (d, *J* = 8.3 Hz, 4H), 4.79–4.67 (m, 2H), 4.61 (s, 2H), 4.37–4.27 (m, 1H), 3.64 (s, 3H), 3.13 (q, *J* = 6.7 Hz, 2H), 1.83–1.64 (m, 2H), 1.51–1.38 (m, *J* = 6.6 Hz, 2H), 1.37–1.22 (m, 2H) (Figure S28). ^13^C NMR (126 MHz, DMSO‐*d*_6_) δ 186.96, 172.29, 167.48, 166.94, 161.90, 161.84, 151.29, 150.57, 138.41, 138.25, 130.99, 130.96, 130.58, 130.24, 124.56, 124.49, 114.78, 66.99, 66.60, 51.93, 51.64, 38.00, 30.25, 28.54, 22.70 (Figure S29). HRMS (ESIMS) calculated for C_39_H_37_N_4_O_8_Cl_2_[M + H]^+^: m/z 759.19830; found: 759.19803 (Figure S30).

Methyl N^2^, N^6^‐bis(2‐(4‐((E)‐3‐(6‐bromopyridin‐3‐yl)acryloyl)phenoxy)acetyl)lysinate **(3k)**. White solid, yield: 78%.^1^H NMR (500 MHz, DMSO‐*d*_6_) δ 8.82 (d, *J* = 2.4 Hz, 2H), 8.32 (dt, *J* = 8.4, 2.2 Hz, 2H), 8.18 (dd, *J* = 8.9, 2.2 Hz, 4H), 8.13 (d, *J* = 4.3 Hz, 1H), 8.09 (d, *J* = 4.3 Hz, 1H), 7.75 (d, *J* = 8.3 Hz, 2H), 7.71 (s, 1H), 7.68 (s, 1H), 7.10 (dd, *J* = 8.9, 1.6 Hz, 4H), 4.77–4.68 (m, 2H), 4.61 (s, 2H), 4.35–4.27 (m, 1H), 3.63 (s, 3H), 3.18–3.08 (m, 2H), 1.81–1.68 (m, 2H), 1.51–1.39 (m, 2H), 1.37–1.24 (m, 2H) (Figure S31). ^13^C NMR (126 MHz, DMSO‐*d*_6_) δ 186.95, 172.27, 167.47, 166.93, 161.90, 161.85, 150.95, 142.58, 138.31, 138.29, 138.05, 138.03, 130.97, 130.93, 130.55, 130.47, 128.20, 124.66, 114.76, 66.98, 66.59, 51.90, 51.65, 37.98, 30.23, 28.50, 22.68 (Figure S32). HRMS (ESIMS) calculated for C_39_H_37_N_4_O_8_Br_2_[M + H]^+^: m/z 847.09727; found: 847.09751 (Figure S33).

Methyl N^2^, N^6^‐bis(2‐(4‐((E)‐3‐(6‐methoxypyridin‐3‐yl)acryloyl)phenoxy)acetyl)lysinate **(3l)**. Pale yellow solid, yield: 60%. ^1^H NMR (500 MHz, Chloroform‐*d*) δ 8.37 (s, 2H), 8.04 (dd, *J* = 8.8, 3.7 Hz, 4H), 7.91 (dd, *J* = 8.7, 2.0 Hz, 2H), 7.78 (s, 1H), 7.75 (s, 1H), 7.45 (d, *J* = 1.8 Hz, 1H), 7.42 (d, *J* = 1.8 Hz, 1H), 7.05–6.99 (m, 5H), 6.80 (d, *J* = 8.7 Hz, 2H), 6.61 (t, *J* = 5.8 Hz, 1H), 4.72–4.68 (m, 1H), 4.59 (d, *J* = 1.4 Hz, 2H), 4.53 (s, 2H), 3.99 (s, 6H), 3.76 (s, 3H), 3.33 (q, *J* = 6.9 Hz, 2H), 1.98–1.89 (m, 1H), 1.81–1.71 (m, 1H), 1.64–1.52 (m, 2H), 1.39–1.28 (m, 2H) (Figure S34). ^13^C NMR (126 MHz, Chloroform‐*d*) δ 188.29, 188.22, 172.34, 167.58, 167.47, 165.58, 160.81, 160.78, 149.13, 141.09, 141.05, 136.71, 136.69, 132.59, 132.51, 131.04, 131.01, 124.42, 120.58, 120.56, 114.82, 114.69, 111.71, 67.45, 67.39, 53.98, 52.71, 51.64, 38.73, 32.04, 29.04, 22.54 (Figure S35). HRMS (ESIMS) calculated for C_41_H_43_N_4_O_10_[M + H]^+^: m/z 751.29737; found: 751.29762 (Figure S36).

### 2.2 In Vitro Antiproliferative Activity Evaluation

All cell lines were purchased from the American Type Culture Collection (ATCC) or FuHeng Biology (China) and authenticated via short tandem repeat (STR) profiling. Huh1, Huh7, and HepG2 cells were cultured in Dulbecco's modified Eagle's medium (DMEM) supplemented with 10% fetal bovine serum (FBS). The cytotoxicity of the synthesized compounds was assessed using the Cell Counting Kit‐8 (CCK‐8) assay.

Briefly, all cell lines were seeded in 96‐well plates and incubated at 37°C in a humidified atmosphere containing 5% CO_2_ for 24 h to allow adherence and stabilization. Subsequently, the cells were treated with various concentrations of the test compounds and further incubated under the same conditions for 48 h. After treatment, the optical density (OD) was measured at a wavelength of 450 nm using a microplate reader. The half‐maximal inhibitory concentration (IC_50_) values were calculated from the dose–response curves using GraphPad Prism 8 software.

### 2.3 In Vitro Live/Dead Cell Assay

To assess the effects of compounds **3d**, **3f**, and the positive control sorafenib on the viability of hepatocellular carcinoma cells, a live/dead cell double‐staining assay (Calcein‐AM/PI) was conducted. Huh1 cells were cultured in DMEM supplemented with 10% FBS until they reached the logarithmic growth phase. Cells were then seeded into 6‐well plates at a density of 5 × 10^5^ cells per well and incubated at 37°C for 24 h to allow for attachment.

Subsequently, cells were treated with compound **3d** (20 μM), compound **3f** (20 μM), or sorafenib (1 μM) for 48 h. Following treatment, the culture medium was aspirated, and cells were washed twice with PBS. A staining solution containing Calcein‐AM and PI was added, and the cells were incubated at 37°C in the dark for 30 min. After staining, the cells were visualized and imaged using a fluorescence microscope.

### 2.4 In Vitro Apoptosis Assay

The capacity of compounds **3d**, **3f**, and the positive control sorafenib to induce apoptosis in Huh1 cells was evaluated using Annexin V‐FITC/PI double staining in conjunction with flow cytometry. For this assay, Huh1 cells were seeded in 6‐well plates at a density of 5 × 10^5^ cells/well and treated with 20 μM compound **3d**, 20 μM compound **3f**, or 1 μM sorafenib for 48 h.

Post‐treatment, cells were harvested and stained following the manufacturer's protocol for Annexin V‐FITC/PI double staining: briefly, cells were incubated with Annexin V‐FITC in the dark for 15 min, after which PI staining was performed on ice for 5 min. Stained cells were subsequently analyzed via flow cytometry.

### 2.5 Western Blot Analysis of Apoptosis Markers

Western blot analysis was performed to assess the expression levels of apoptosis‐related proteins in Huh1 cells after treatment with compounds **3d**, **3f**, and the positive control sorafenib. Briefly, Huh1 cells were treated with 20 μM compound **3d**, 20 μM compound **3f**, or 1 μM sorafenib for 48 h.

Total proteins were extracted using RIPA lysis buffer supplemented with protease inhibitors, and protein concentrations were determined via the BCA (bicinchoninic acid) assay. Equal amounts of protein samples were separated by 10% SDS‐PAGE (sodium dodecyl sulfate‐polyacrylamide gel electrophoresis) and subsequently transferred onto PVDF (polyvinylidene difluoride) membranes. After blocking with 5% non‐fat milk in TBST (Tris‐buffered saline with Tween 20) for 1 h at room temperature, the membranes were incubated overnight at 4°C with primary antibodies specific to Caspase‐3, Cleaved Caspase‐3, and GAPDH (glyceraldehyde‐3‐phosphate dehydrogenase). Following three washes with TBST, the membranes were incubated with corresponding HRP (horseradish peroxidase)‐conjugated secondary antibodies for 1 h at room temperature, and protein signals were detected using chemiluminescence.

## 3 Results and Discussion

### 3.1 Chemistry

**SCHEME 1:**
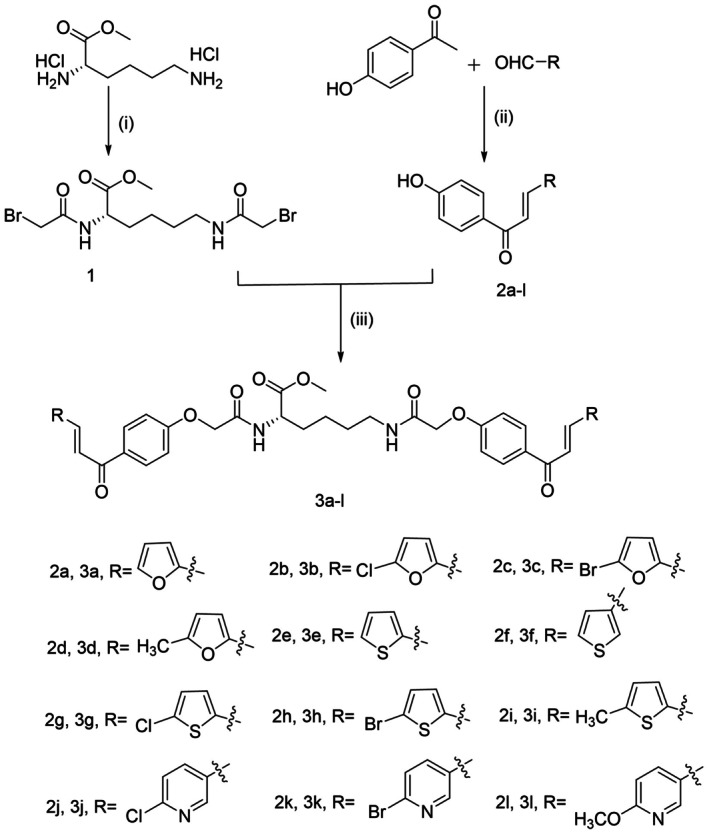
General synthetic scheme of **3a‐l**. Reagents and conditions: (i) 2‐bromoacetyl bromide, Na_2_CO_3_, NaHCO_3_, CH_2_Cl_2_, H_2_O, −5°C; (ii) NaOH, C_2_H_5_OH, room temperature; (iii) K_2_CO_3_, DMF, 60°C.

In this work, a series of heterocyclic chalcones containing furan, thiophene, and pyridine moieties were synthesized via Claisen‐Schmidt condensation reactions between 4‐hydroxyacetophenone and various substituted heterocyclic aldehydes. Two molecules of heterocyclic chalcone were further linked using modified lysine to yield bis‐heterocyclic chalcones with yields ranging from 50% to 78%. The structures of the target compounds were confirmed by nuclear magnetic resonance spectroscopy (^1^H NMR, ^13^C NMR) and high‐resolution mass spectrometry (HRMS). The synthetic route for the lysine‐based bis‐heterocyclic chalcones (**3a‐l**) is depicted in Scheme 1.

### 3.2 In Vitro Antiproliferative Activity

The antiproliferative activity of bis‐heterocyclic chalcones **3a‐l** against a panel of human tumor cell lines was evaluated, with the results summarized in Table 1. Compounds **3d**, **3f**, and **3l** exhibited inhibitory activity against Huh1 cells, with IC_50_ values of 20.91 μM, 6.75 μM, and 7.09 μM, respectively. Among the tested compounds, **3a**, **3d**, **3e**, **3f**, and **3l** were active against Huh‐7 cells, displaying IC_50_ values of 14.16 μM, 9.63 μM, 16.38 μM, 8.40 μM, and 13.24 μM, respectively. Additionally, compound **3d** showed an inhibitory effect on HepG2 cells, with an IC_50_ value of 27.99 μM.

**TABLE 1:** Cytotoxicity of compounds **3a‐l**.

Cell line/Treatment	Huh 1 (IC_50_)	Huh 7 (IC_50_)	HepG2 (IC_50_)
**3a**	> 100 μM	14.16 μM	> 100 μM
**3b**	> 100 μM	> 100 μM	> 100 μM
**3c**	> 100 μM	> 100 μM	> 100 μM
**3d**	20.91 μM	9.63 μM	27.99 μM
**3e**	> 100 μM	16.38 μM	> 100 μM
**3f**	6.75 μM	8.40 μM	> 100 μM
**3g**	> 100 μM	> 100 μM	> 100 μM
**3h**	> 100 μM	> 100 μM	> 100 μM
**3i**	> 100 μM	> 100 μM	> 100 μM
**3j**	> 100 μM	> 100 μM	> 100 μM
**3k**	> 100 μM	> 100 μM	> 100 μM.
**3l**	7.09 μM	13.24 μM	> 100 μM
**sorafenib**	15.50 μM	15.34 μM	8.88 μM

*Note:* IC_50_ is the concentration of compound needed to reduce cell growth by 50% following 48 h cell treatment with the tested drugs.

### 3.3 Investigating the Antitumor Mechanisms of Compounds **3d** and **3f**

**FIGURE 2:**
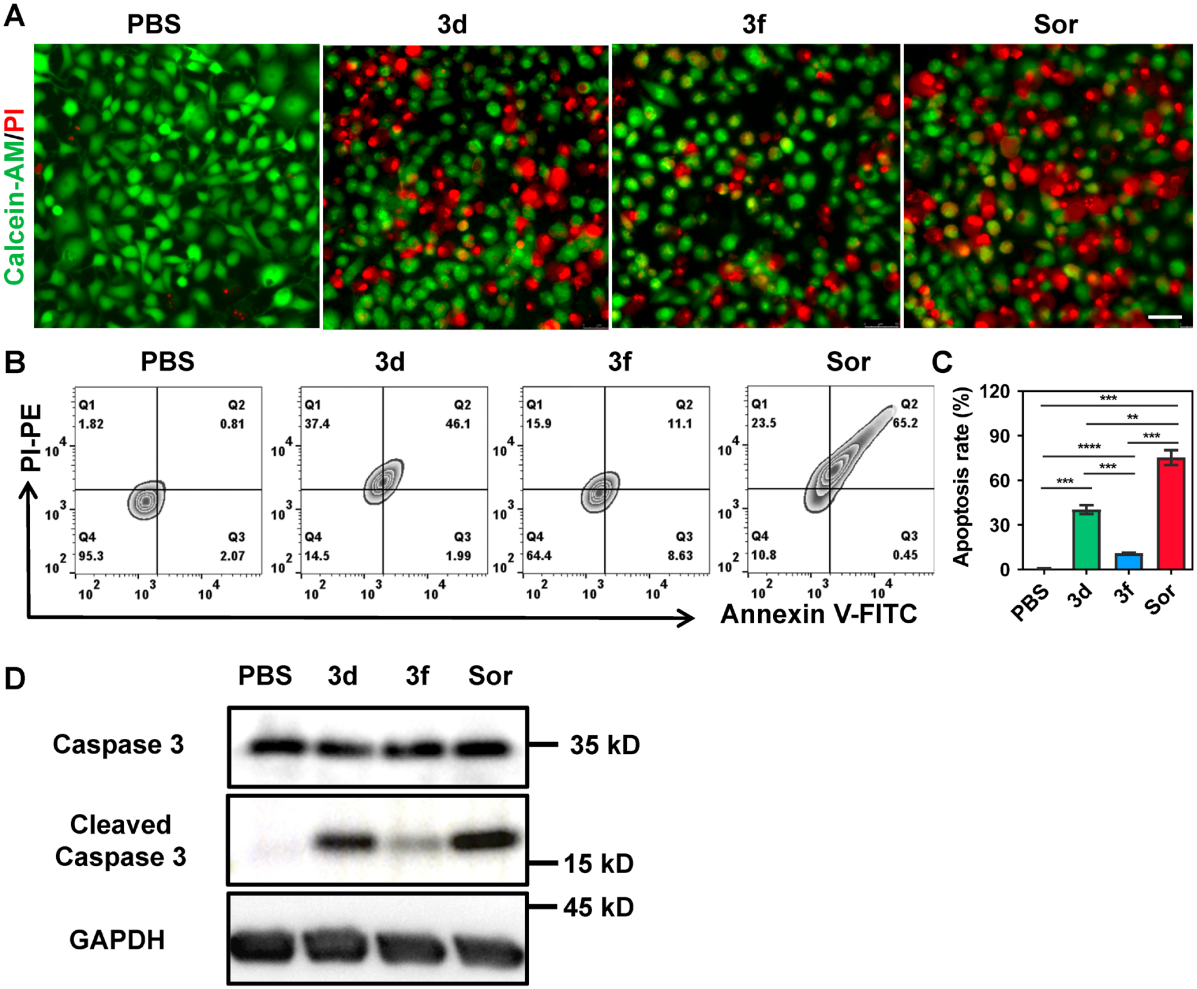
(A) Live/dead cell analysis after different treatments in Huh1 cells through calcein‐AM (green) and PI (red) staining. Scale bars: 50 μm. (B) Apoptosis detection of Huh1 cells after various treatments by flow cytometric analysis. (C) Quantification of apoptosis in Huh1 cells after various treatments by flow‐cytometry analysis. Data are presented as mean ± S.D. (*n* = 3, *p < 0.05, **p < 0.01, ***p < 0.001, ****p < 0.0001, two‐tailed student's *t*‐test). (D) Western blotting analysis of Caspase 3 and Cleaved‐caspase 3 in Huh1 cells after various treatments.

Based on the CCK‐8 assay results, compounds **3d** and **3f** were further evaluated in Huh‐1 cells using multiple assays to explore their potential antitumor mechanisms. First, live/dead cell assessment was performed via calcein‐AM and propidium iodide (PI) dual staining (Figure 2A), with sorafenib serving as a positive control. The results demonstrated that both **3d** and **3f** (20 μM) significantly induced cell death (PI‐positive, red staining), with **3d** exhibiting a more pronounced effect. Notably, 1 μM sorafenib also elicited evident cell death, indicating its strong efficacy in inducing cell death (Figure 2A).

To further determine whether **3d** and **3f** induce cell death through apoptosis, flow cytometry with Annexin V/PI dual staining was conducted. The results showed that both compounds caused significant cell apoptosis compared with the vehicle control (Figure 2B,C). Sorafenib, as a positive control, displayed the most marked apoptotic effect, consistent with its potent tumor‐inhibitory activity as a clinically approved drug for liver cancer (Figure 2B,C).

To investigate the underlying apoptotic mechanisms, the expression levels of caspase‐3 and cleaved caspase‐3 were examined via western blot analysis. Treatment with the three drugs (**3d**, **3f**, and sorafenib) led to upregulated expression of cleaved caspase‐3 in Huh‐1 cells (Figure 2D), with **3d** and sorafenib showing more significant changes, which is consistent with the flow cytometry results.

## 4 Discussion

Structure–activity relationship (SAR) analysis revealed that the introduction of an electron‐donating methyl group on the furan ring (compound **3d**) resulted in inhibitory activity against Huh‐1, Huh‐7, and HepG2 cell lines. In contrast, both electron‐donating and electron‐withdrawing substituents on the thiophene ring were found to diminish the inhibitory potency against Huh‐1 and Huh‐7 cells. Notably, the presence of an electron‐donating methoxy group on the pyridine ring (compound **3l**) was associated with potent inhibitory effects on both Huh‐1 and Huh‐7 cells.

In conclusion, twelve bis‐heterocyclic chalcones were successfully synthesized under mild reaction conditions via the alkylation of modified lysine with diverse heterocyclic chalcones. Their in vitro biological activities were assessed against Huh1, Huh7, and HepG2 cell lines. The results indicated that compounds **3d**, **3f** and **3l** exerted the highest activity, with IC_50_ values ranging from 6.75 to 20.91 μM against Huh1 and Huh7 cells. Moreover, compound **3d** emerged as the most potent cytotoxic agent against HepG2 cells, with an IC_50_ value of 27.99 μM. Furthermore, collective data from live/dead cell staining, flow cytometric analysis, and Western blotting provide compelling evidence that compounds **3d** and **3f** effectively induce apoptosis in hepatocellular carcinoma cells.

## Conflicts of Interest

The authors declare no conflicts of interest.

## Supporting information


**Figures S1‐S36:** jcla70154‐sup‐0001‐FiguresS1‐S36.docx.
**Figure S1:** ^1^H NMR spectrum of compound **3a** in Chloroform‐*d*.
**Figure S2:** ^13^C NMR spectrum of compound **3a** in Chloroform‐*d*.
**Figure S3:** HRMS of compound **3a**.
**Figure S4:** ^1^H NMR spectrum of compound **3b** in Chloroform‐*d*.
**Figure S5:** ^13^C NMR spectrum of compound **3b** in Chloroform‐*d*.
**Figure S6:** HRMS of compound **3b**.
**Figure S7:** ^1^H NMR spectrum of compound **3c** in Chloroform‐*d*.
**Figure S8:** ^13^C NMR spectrum of compound **3c** in Chloroform‐*d*.
**Figure S9:** HRMS of compound **3c**.
**Figure S10:** ^1^H NMR spectrum of compound **3d** in DMSO‐*d*_6_.
**Figure S11:** ^13^C NMR spectrum of compound **3d** in DMSO‐*d*_6_.
**Figure S12:** HRMS of compound **3d**.
**Figure S13:** ^1^H NMR spectrum of compound **3e** in Chloroform‐*d*.
**Figure S14:** ^13^C NMR spectrum of compound **3e** in Chloroform‐*d*.
**Figure S15:** HRMS of compound **3e**.
**Figure S16:** ^1^H NMR spectrum of compound **3f** in Chloroform‐*d*.
**Figure S17:** ^13^C NMR spectrum of compound **3f** in Chloroform‐*d*.
**Figure S18:** HRMS of compound **3f**.
**Figure S19:** ^1^H NMR spectrum of compound **3g** in Chloroform‐*d*.
**Figure S20:** ^13^C NMR spectrum of compound **3g** in Chloroform‐*d*.
**Figure S21:** HRMS of compound **3g**.
**Figure S22:** ^1^H NMR spectrum of compound **3h** in Chloroform‐*d*.
**Figure S23:** ^13^C NMR spectrum of compound **3h** in Chloroform‐*d*.
**Figure S24:** HRMS of compound **3h**.
**Figure S25:** ^1^H NMR spectrum of compound **3i** in Chloroform‐*d*.
**Figure S26:** ^13^C NMR spectrum of compound **3i** in Chloroform‐*d*.
**Figure S27:** HRMS of compound **3i**.
**Figure S28:** ^1^H NMR spectrum of compound **3j** in DMSO‐*d*_6_.
**Figure S29:** ^13^C NMR spectrum of compound **3j** in DMSO‐*d*_6_.
**Figure S30:** HRMS of compound **3j**.
**Figure S31:** ^1^H NMR spectrum of compound **3k** in DMSO‐*d*_6_.
**Figure S32:** ^13^C NMR spectrum of compound **3k** in DMSO‐*d*_6_.
**Figure S33:** HRMS of compound **3k**.
**Figure S34:** ^1^H NMR spectrum of compound **3l** in Chloroform‐*d*.
**Figure S35:** ^13^C NMR spectrum of compound **3l** in Chloroform‐*d*.
**Figure S36:** HRMS of compound **3l**.

## Data Availability

The data that supports the findings of this study are available in the Supporting Information of this article.
