# Supplementary material for: Synthesis, Antitumor Activity Evaluation and Mechanistic Study of Novel Bis‐Heterocyclic Chalcones Against Liver Cancer
Source: J Clin Lab Anal. 2026 Feb 9;40(5):e70154. doi: 10.1002/jcla.70154 (PMC13042619; doi:10.1002/jcla.70154)
Supplement: Supplementary file 1 — Figures S1‐S36: jcla70154‐sup‐0001‐FiguresS1‐S36.docx. Figure S1: 1H NMR spectrum of compound 3a in Chloroform‐d. Figure S2: 13C NMR spectrum of compound 3a in Chloroform‐d. Figure S3: HRMS of compound 3a. Figure S4: 1H NMR spectrum of compound 3b in Chloroform‐d. Figure S5: 13C NMR spectrum of compound 3b in Chloroform‐d. Figure S6: HRMS of compound 3b. Figure S7: 1H NMR spectrum of compound 3c in Chloroform‐d. Figure S8: 13C NMR spectrum of compound 3c in Chloroform‐d. Figure S9: HRMS of compound 3c. Figure S10: 1H NMR spectrum of compound 3d in DMSO‐d 6. Figure S11: 13C NMR spectrum of compound 3d in DMSO‐d 6. Figure S12: HRMS of compound 3d. Figure S13: 1H NMR spectrum of compound 3e in Chloroform‐d. Figure S14: 13C NMR spectrum of compound 3e in Chloroform‐d. Figure S15: HRMS of compound 3e. Figure S16: 1H NMR spectrum of compound 3f in Chloroform‐d. Figure S17: 13C NMR spectrum of compound 3f in Chloroform‐d. Figure S18: HRMS of compound 3f. Figure S19: 1H NMR spectrum of compound 3g in Chloroform‐d. Figure S20: 13C NMR spectrum of compound 3g in Chloroform‐d. Figure S21: HRMS of compound 3g. Figure S22: 1H NMR spectrum of compound 3h in Chloroform‐d. Figure S23: 13C NMR spectrum of compound 3h in Chloroform‐d. Figure S24: HRMS of compound 3h. Figure S25: 1H NMR spectrum of compound 3i in Chloroform‐d. Figure S26: 13C NMR spectrum of compound 3i in Chloroform‐d. Figure S27: HRMS of compound 3i. Figure S28: 1H NMR spectrum of compound 3j in DMSO‐d 6. Figure S29: 13C NMR spectrum of compound 3j in DMSO‐d 6. Figure S30: HRMS of compound 3j. Figure S31: 1H NMR spectrum of compound 3k in DMSO‐d 6. Figure S32: 13C NMR spectrum of compound 3k in DMSO‐d 6. Figure S33: HRMS of compound 3k. Figure S34: 1H NMR spectrum of compound 3l in Chloroform‐d. Figure S35: 13C NMR spectrum of compound 3l in Chloroform‐d. Figure S36: HRMS of compound 3l. [file JCLA-40-e70154-s001.docx]

**Supporting Information**

**Synthesis, Antitumor Activity Evaluation and Mechanistic Study of Novel Bis-Chalcones Against Liver Cancer**

Zhifen Li^1,^*^,#^, Jingbo Ma^2,#^, Xiannian Lv^3^, Zhang Lei^1^, Xie Hai^1,^*

*^1^ School of Chemistry and Chemical Engineering, Shanxi Datong University, Xing Yun Street, Pingcheng District, Datong, Shanxi Province 037009, P. R. China*

*^2^Department of Geriatrics, Shenzhen People’s Hospital (The Second Clinical Medical College, Jinan University; The First Affiliated Hospital, Southern University of Science and Technology), Shenzhen, Guangdong 518020, P. R. China*

*^3^ Department of Geriatrics, Fifth People's Hospital of Datong City, Shanxi Province 2669 Wenxing Road North, Pingcheng District, Datong City, 037006, Shanxi, P. R. China*

*Corresponding authors:

[lizhifen_1982@163.com](mailto:lizhifen_1982@163.com) (Zhifen Li);

[xiehai10@126.com](mailto:xiehai10@126.com)(Hai Xie);

# These authors contribute equally to this work and share the first-authorship.

**1. Spectra of the compounds synthesized**


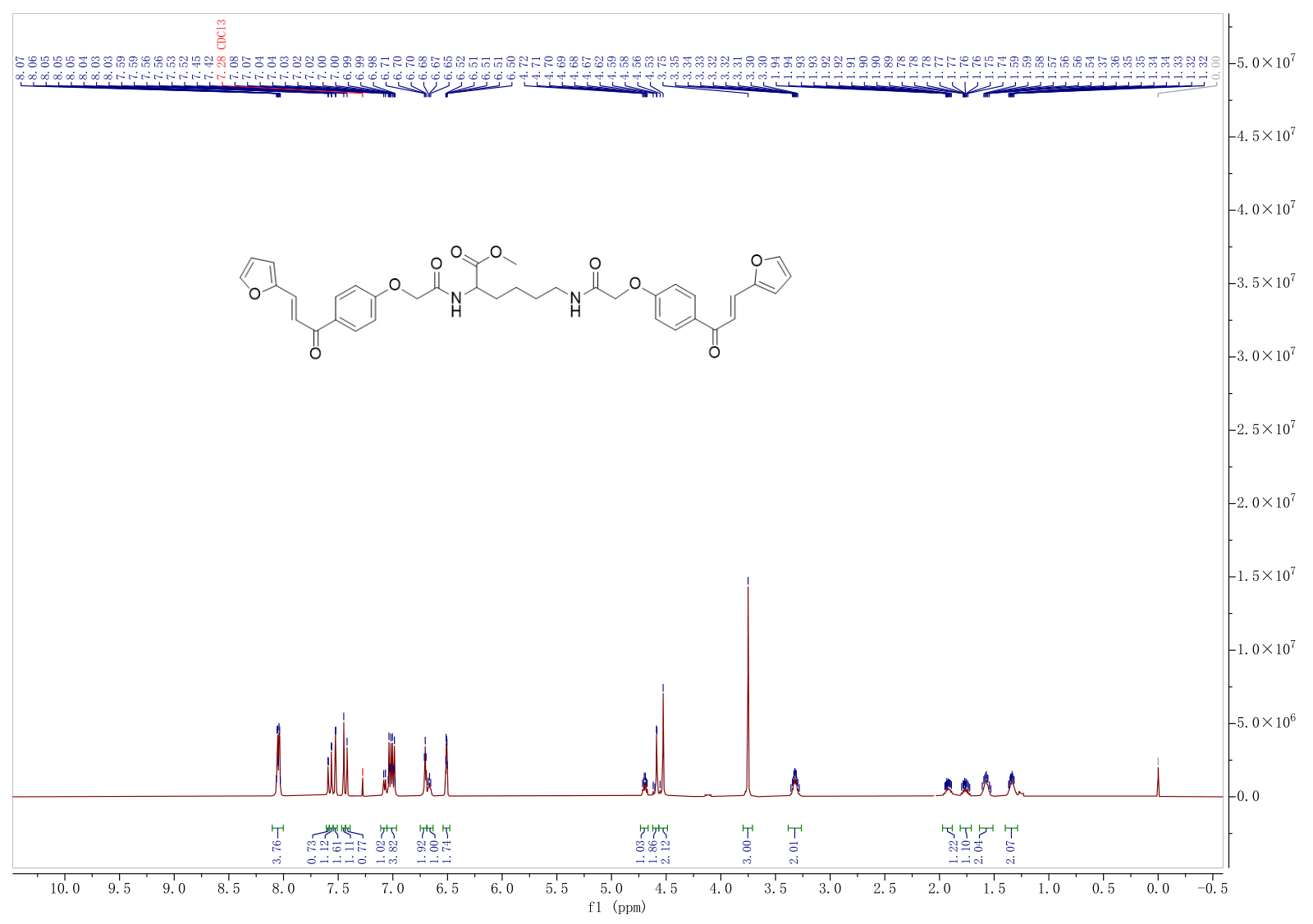


**Fig. S1.** ^1^H NMR spectrum of compound **3a** in Chloroform-*d*.


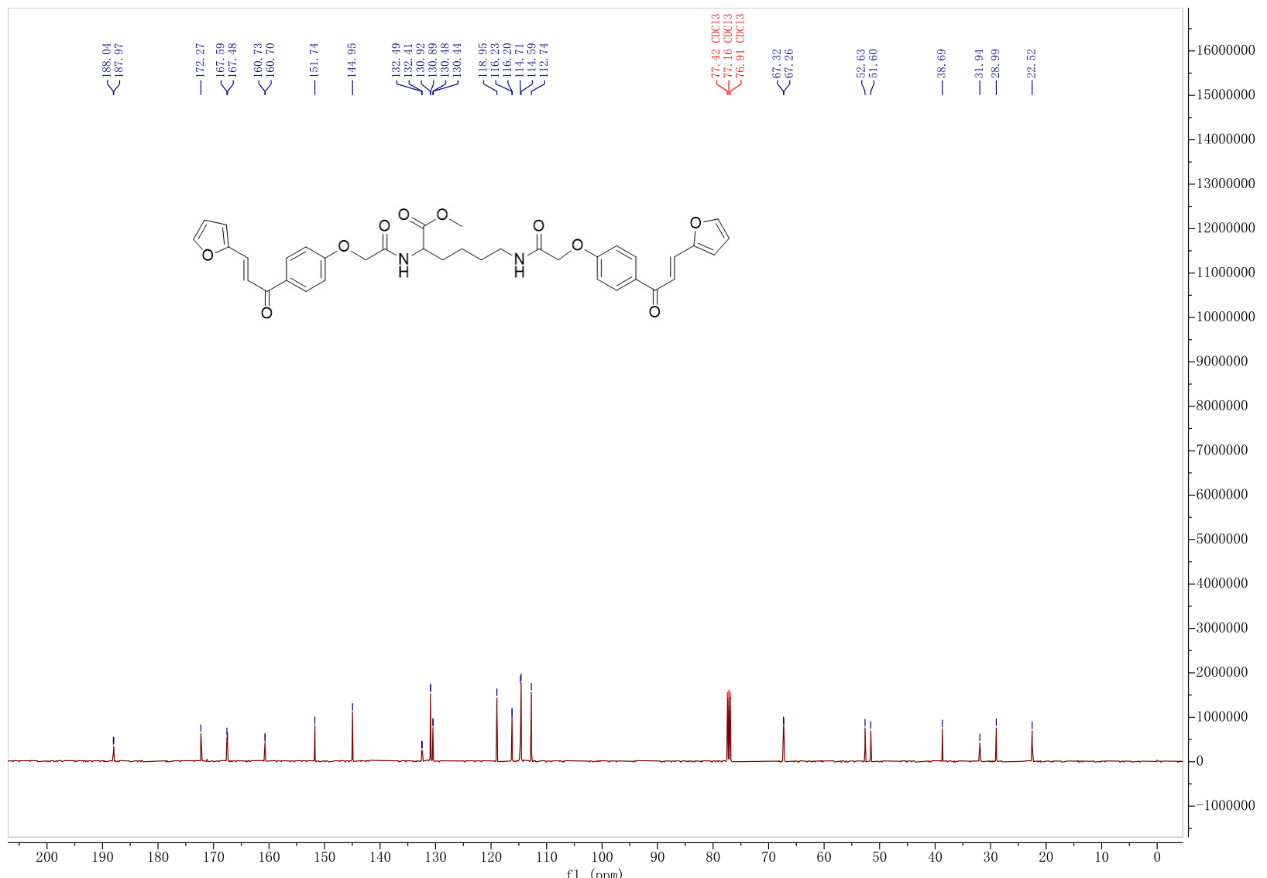


**Fig. S2.** ^13^C NMR spectrum of compound **3a** in Chloroform-*d*.


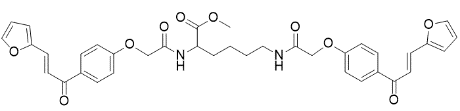

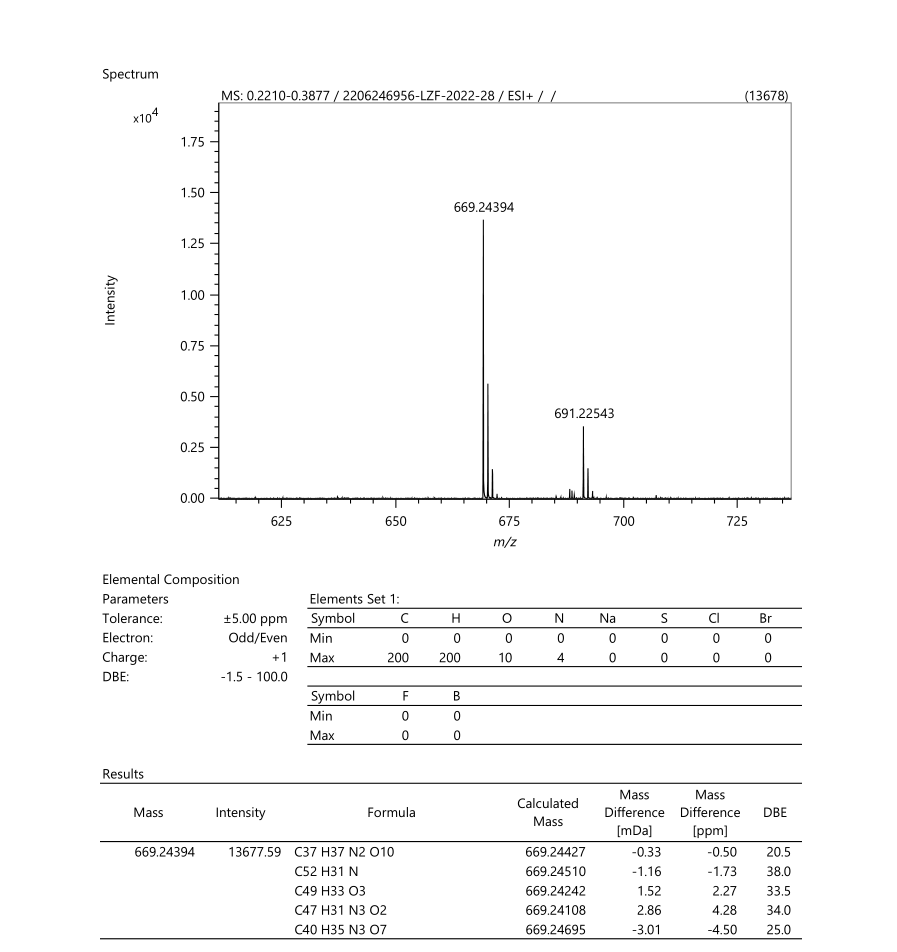


**Fig. S3.** HRMS of compound **3a**.


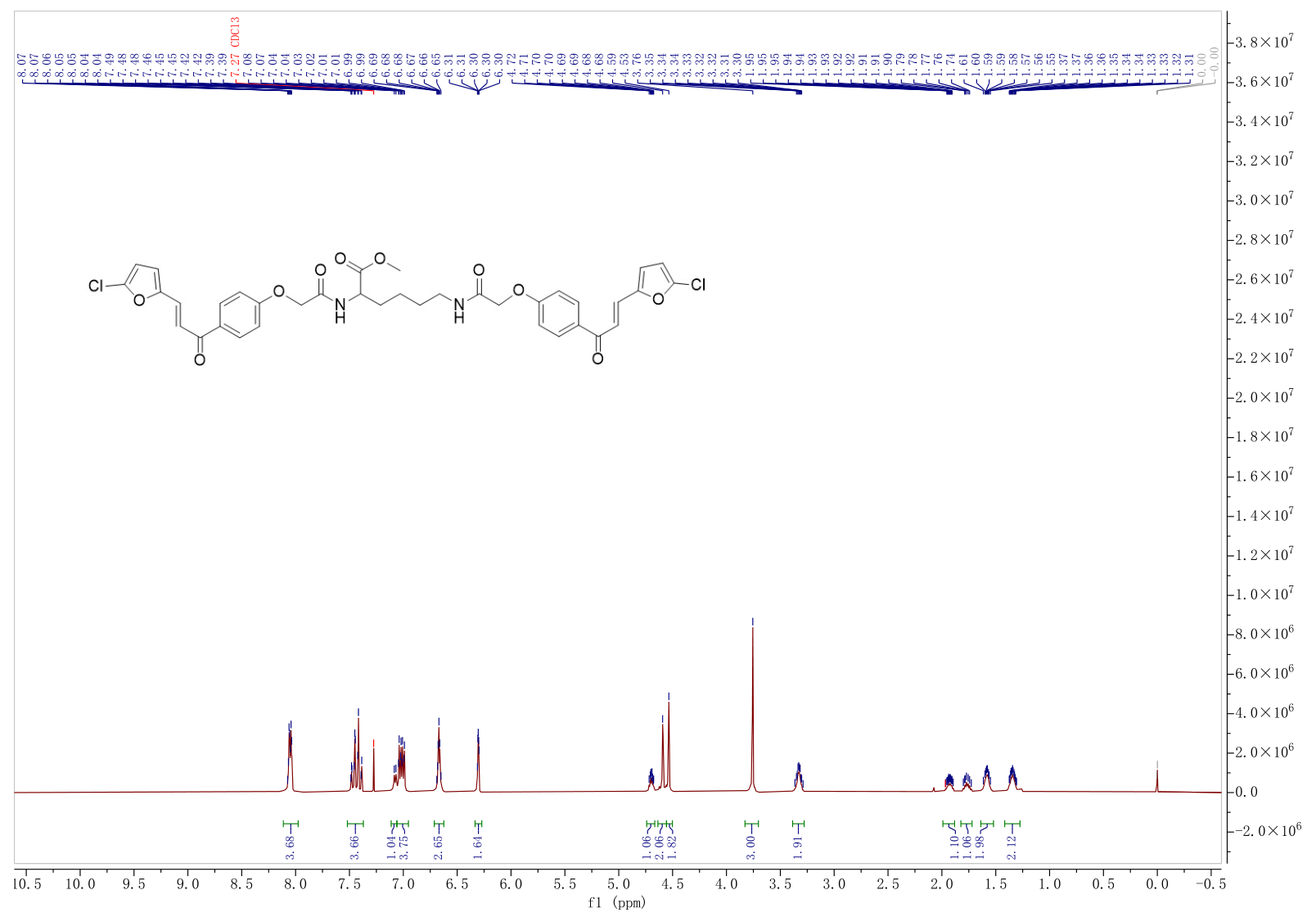


**Fig. S4.** ^1^H NMR spectrum of compound **3b** in Chloroform-*d*.


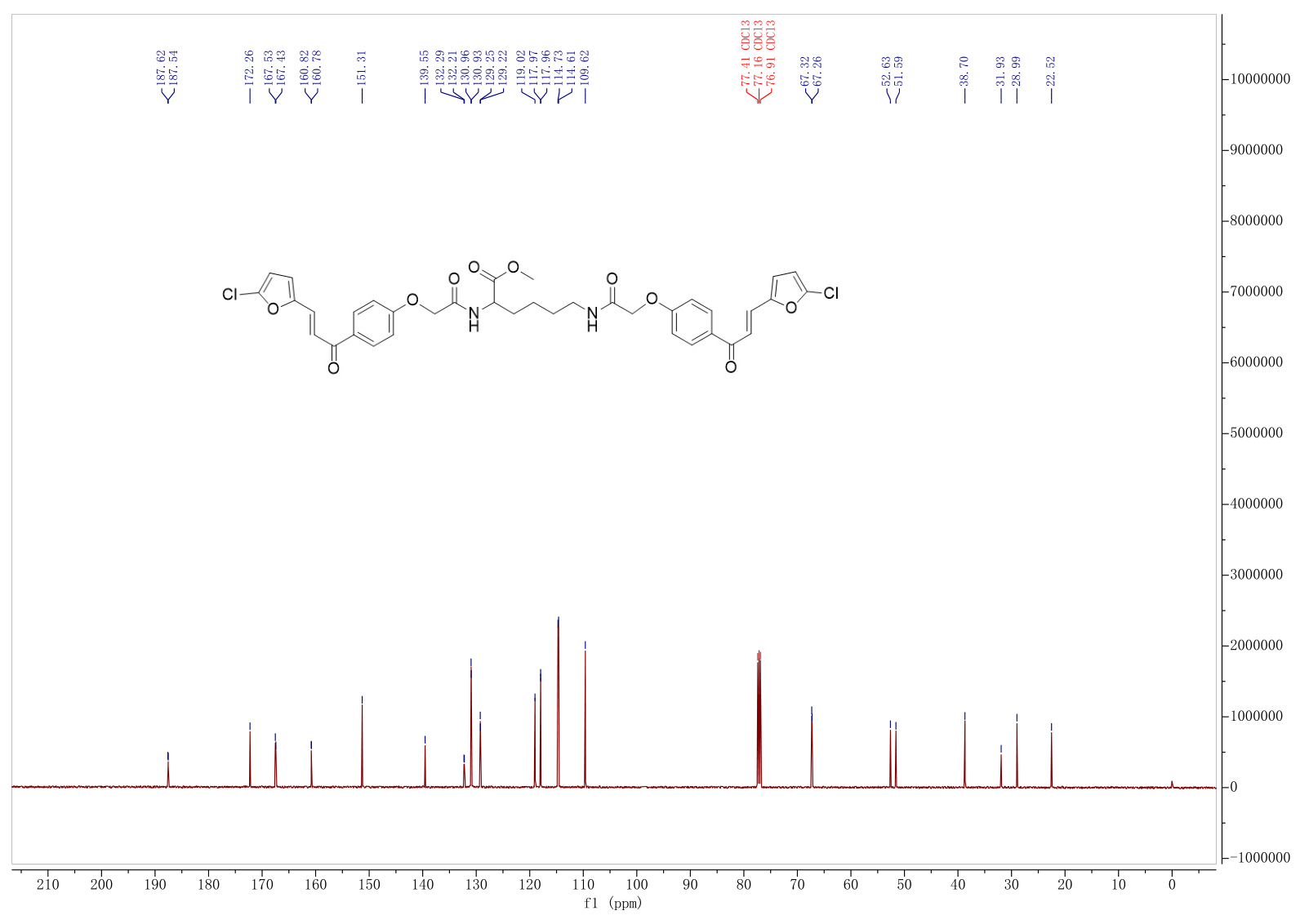


**Fig. S5.** ^13^C NMR spectrum of compound **3b** in Chloroform-*d*.


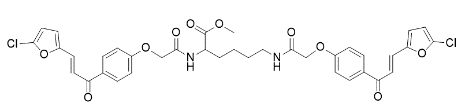

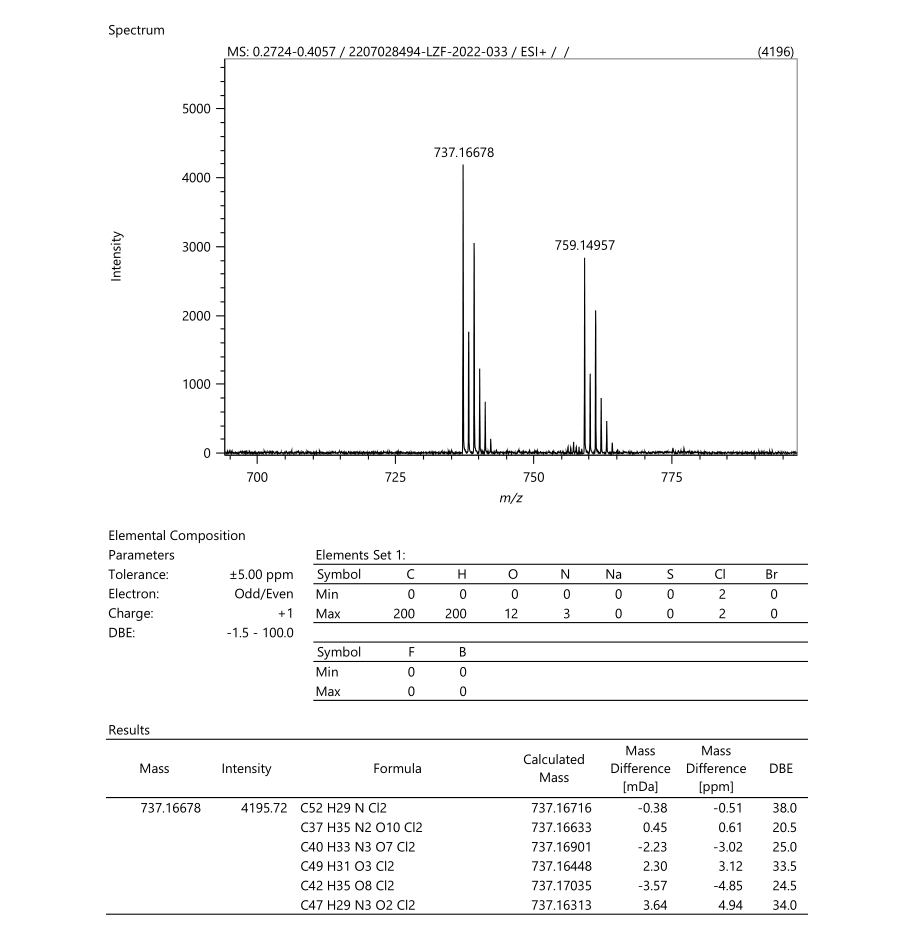


**Fig. S6.** HRMS of compound **3b**.


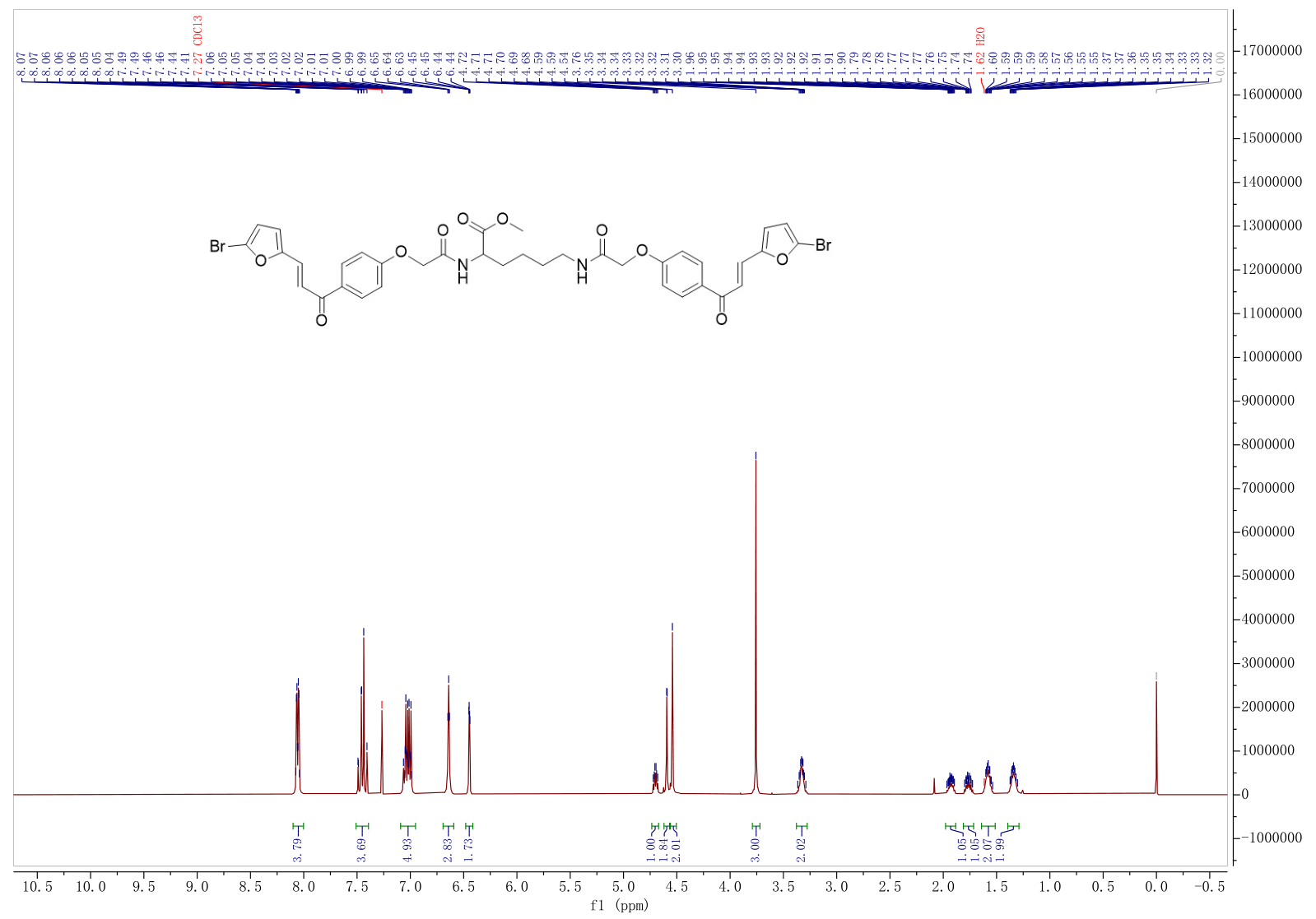


**Fig. S7.** ^1^H NMR spectrum of compound **3c** in Chloroform-*d*.


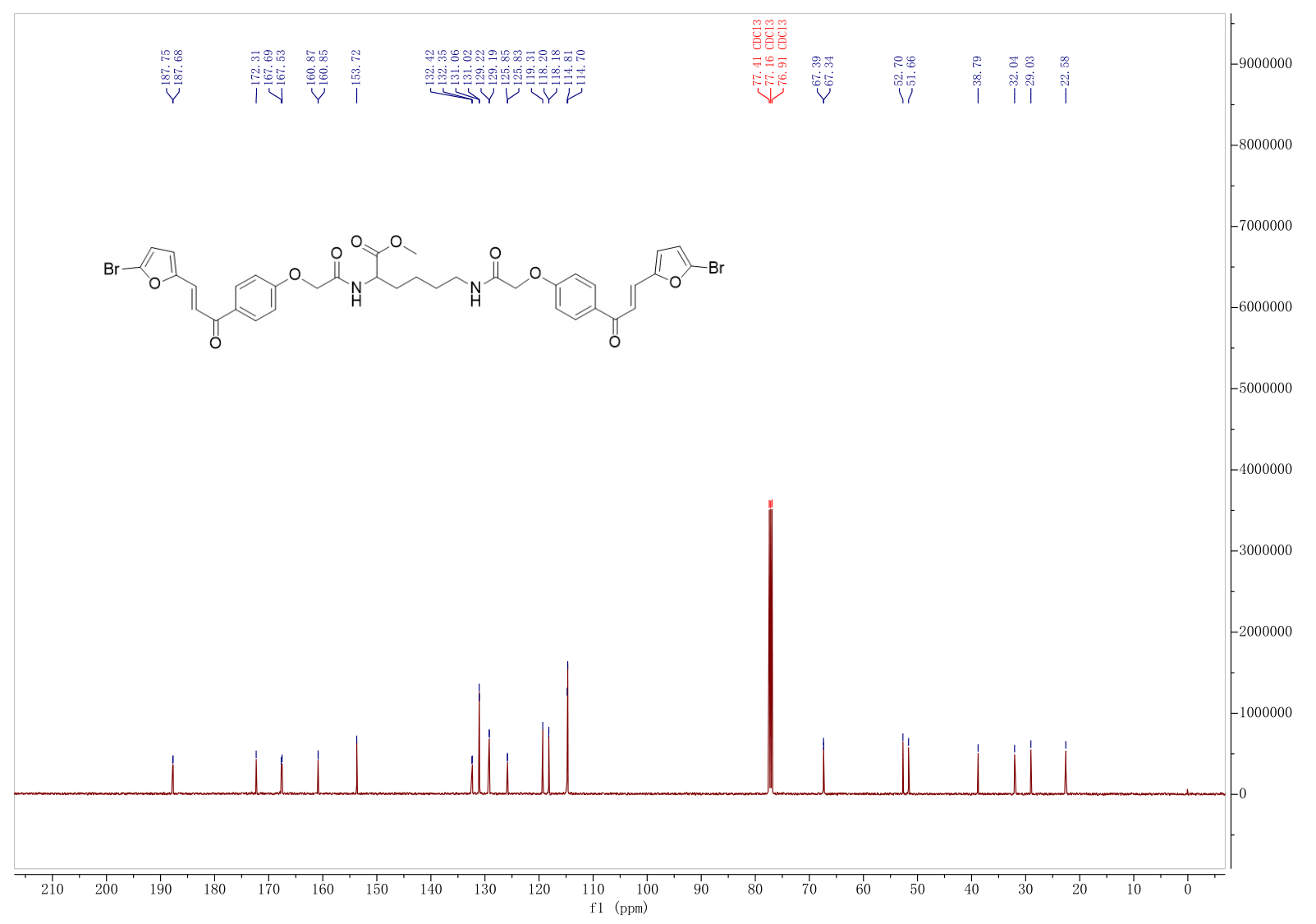


**Fig. S8.** ^13^C NMR spectrum of compound **3c** in Chloroform-*d*.


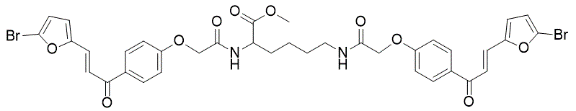

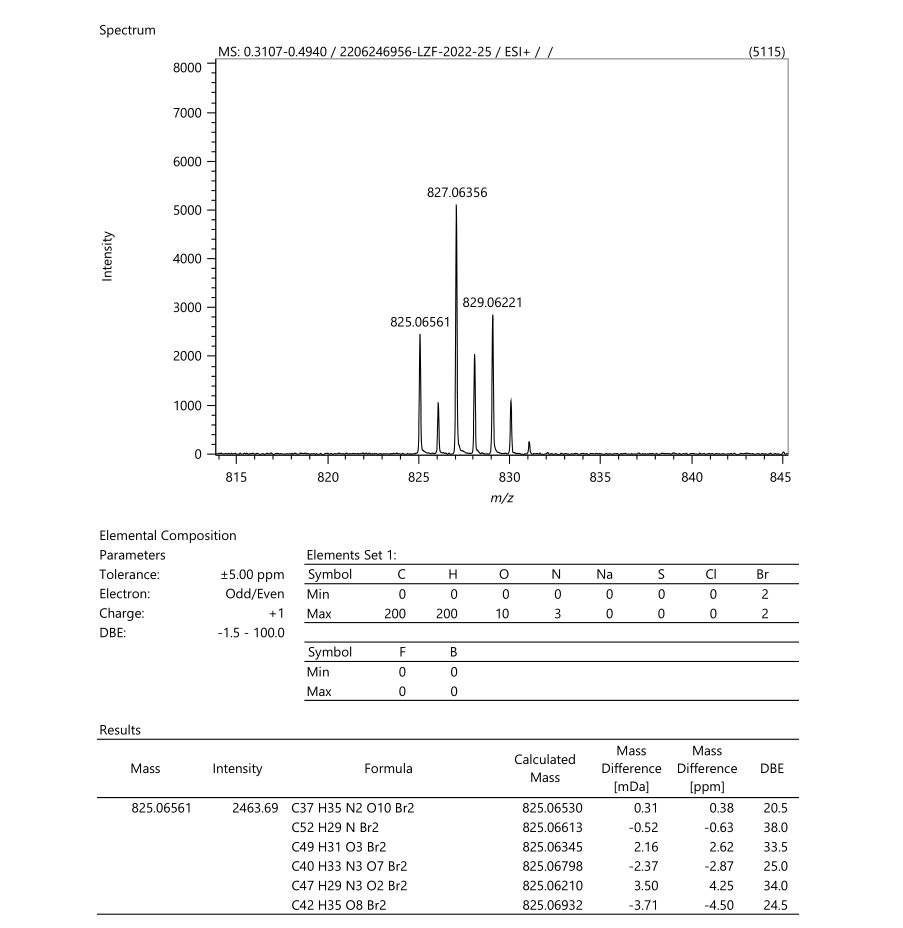


**Fig. S9.** HRMS of compound **3c**.


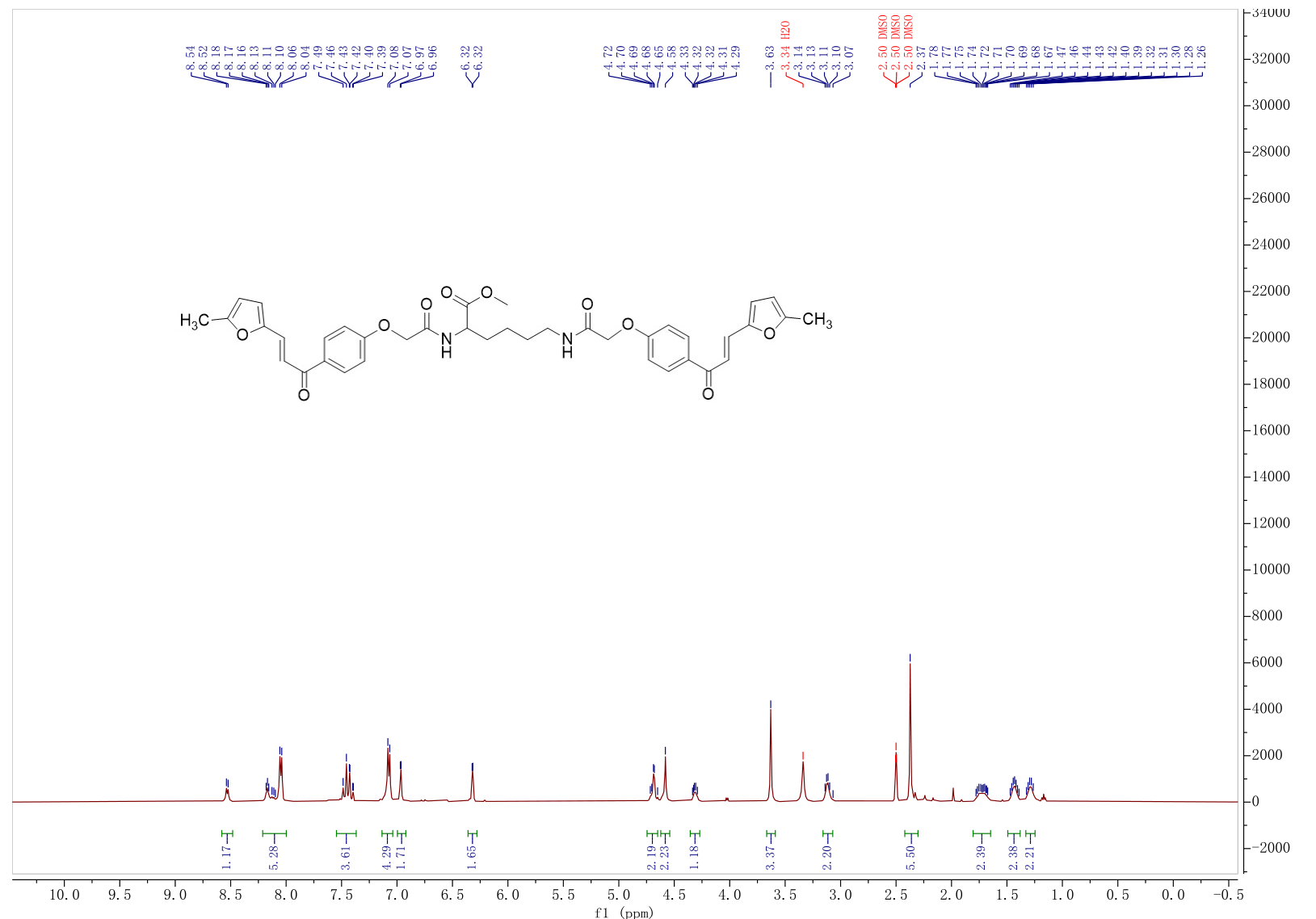


**Fig. S10.** ^1^H NMR spectrum of compound **3d** in DMSO-*d*_6_.


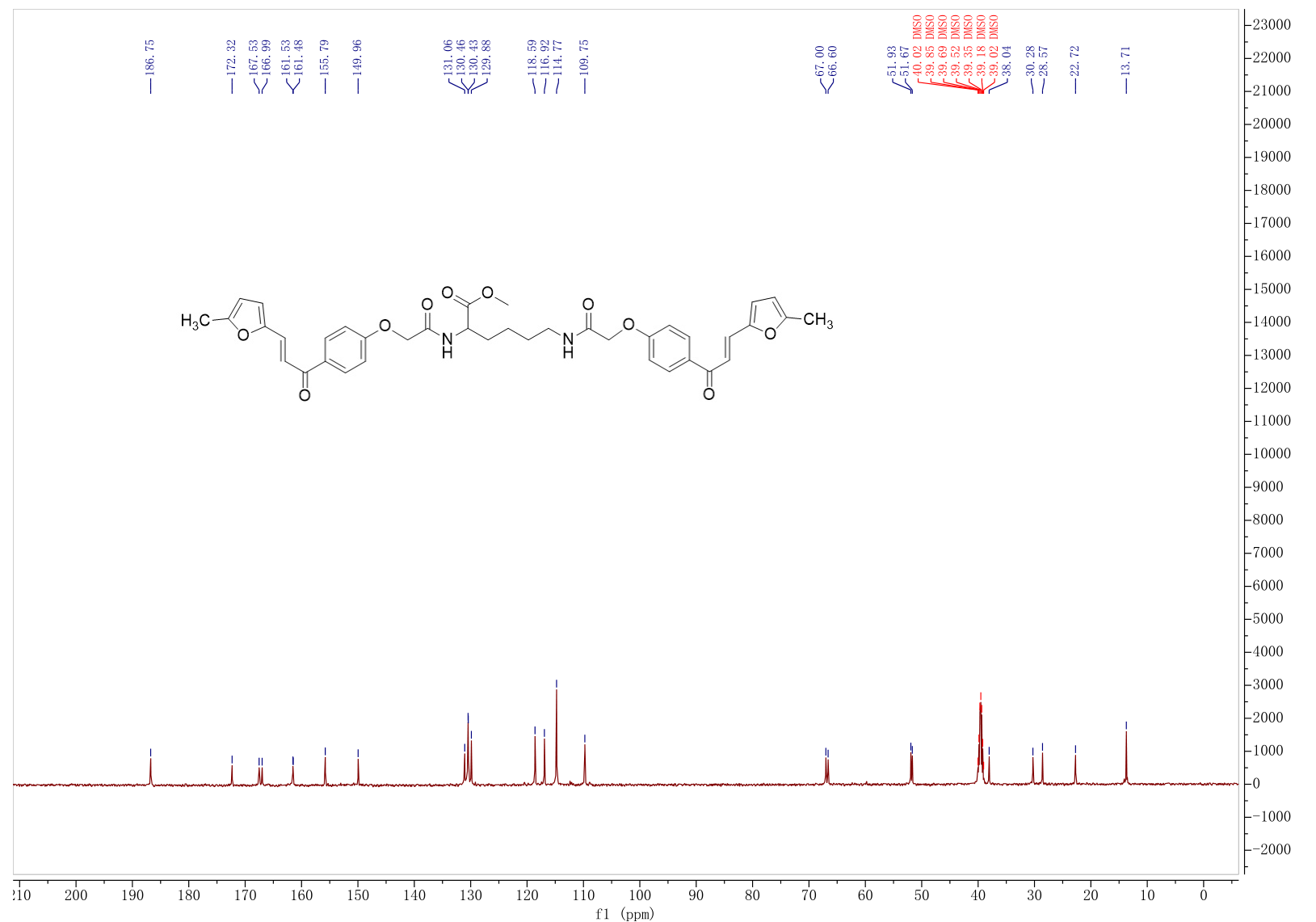


**Fig. S11.** ^13^C NMR spectrum of compound **3d** in DMSO-*d*_6_.


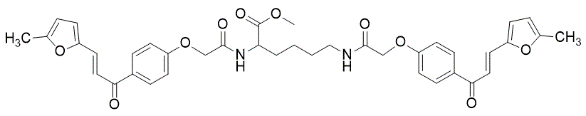

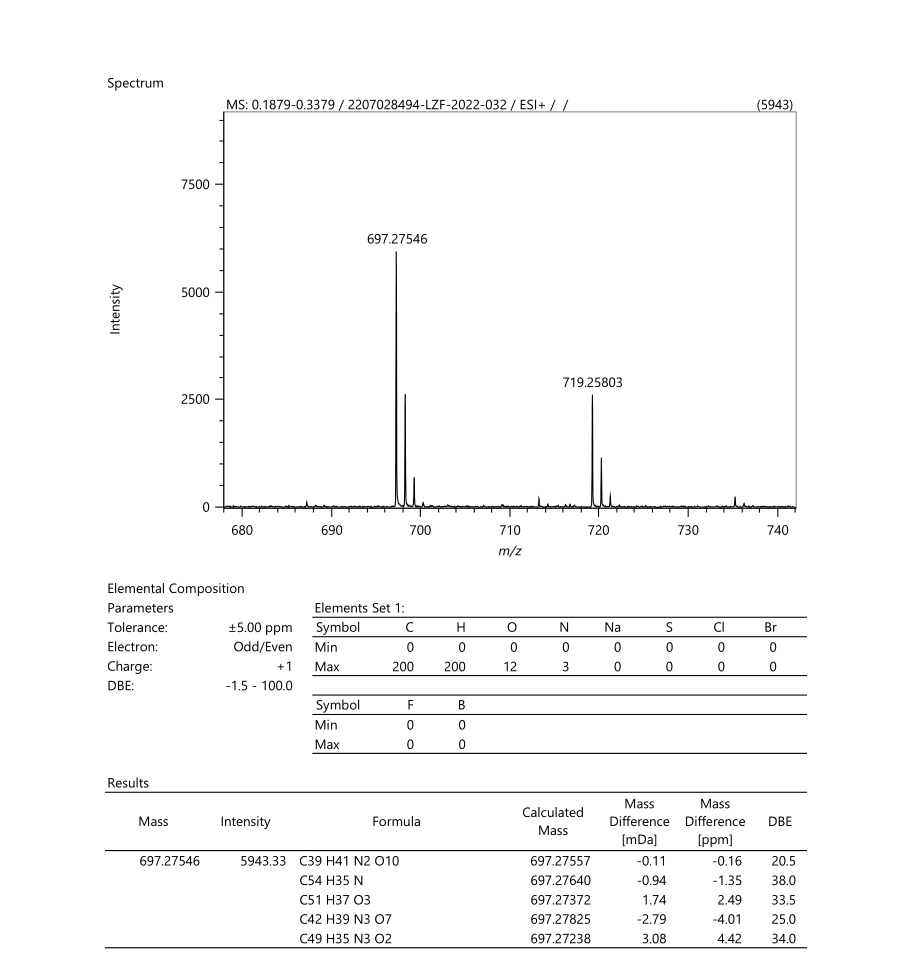


**Fig. S12.** HRMS of compound **3d**.


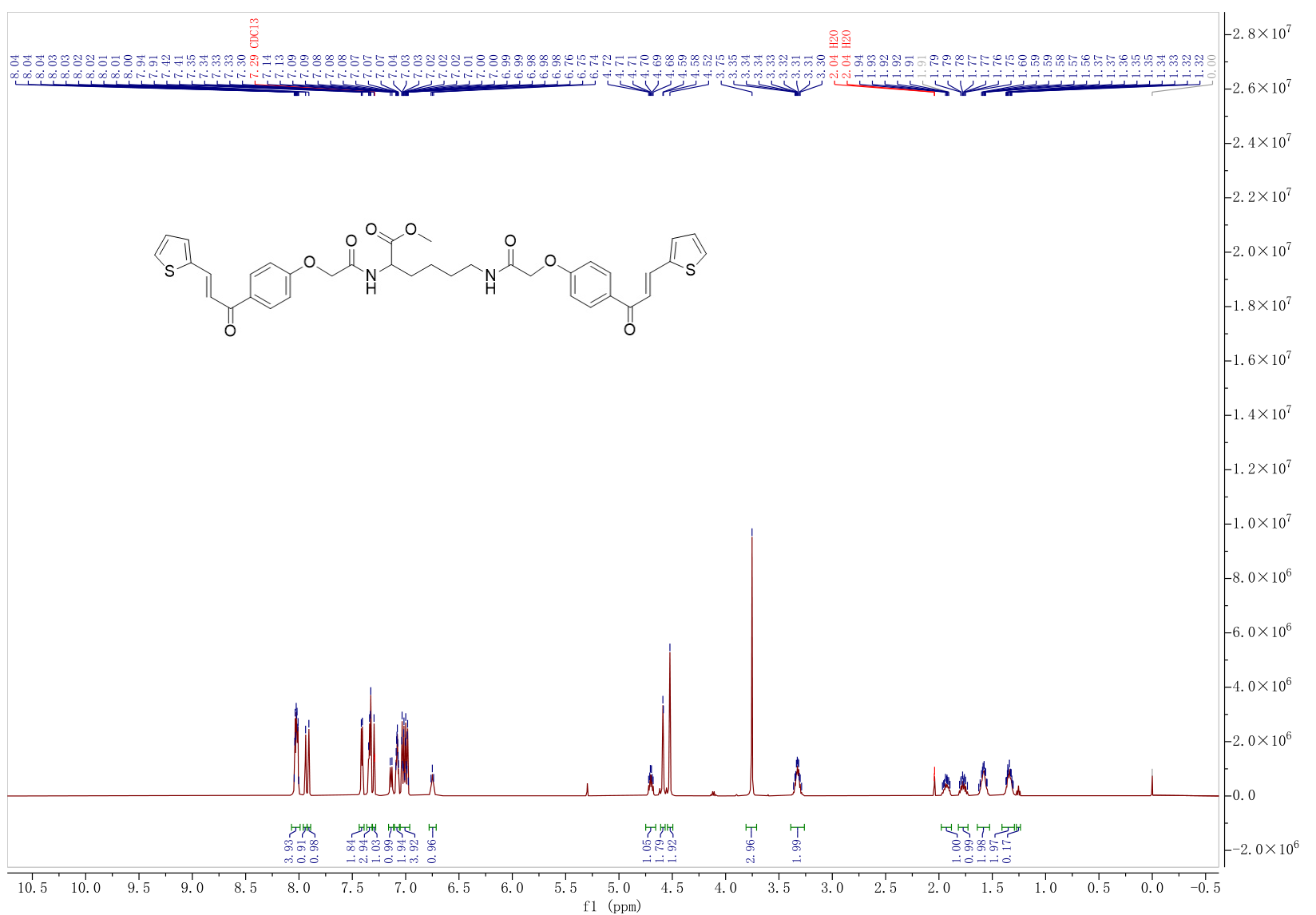


**Fig. S13.** ^1^H NMR spectrum of compound **3e** in Chloroform-*d*.


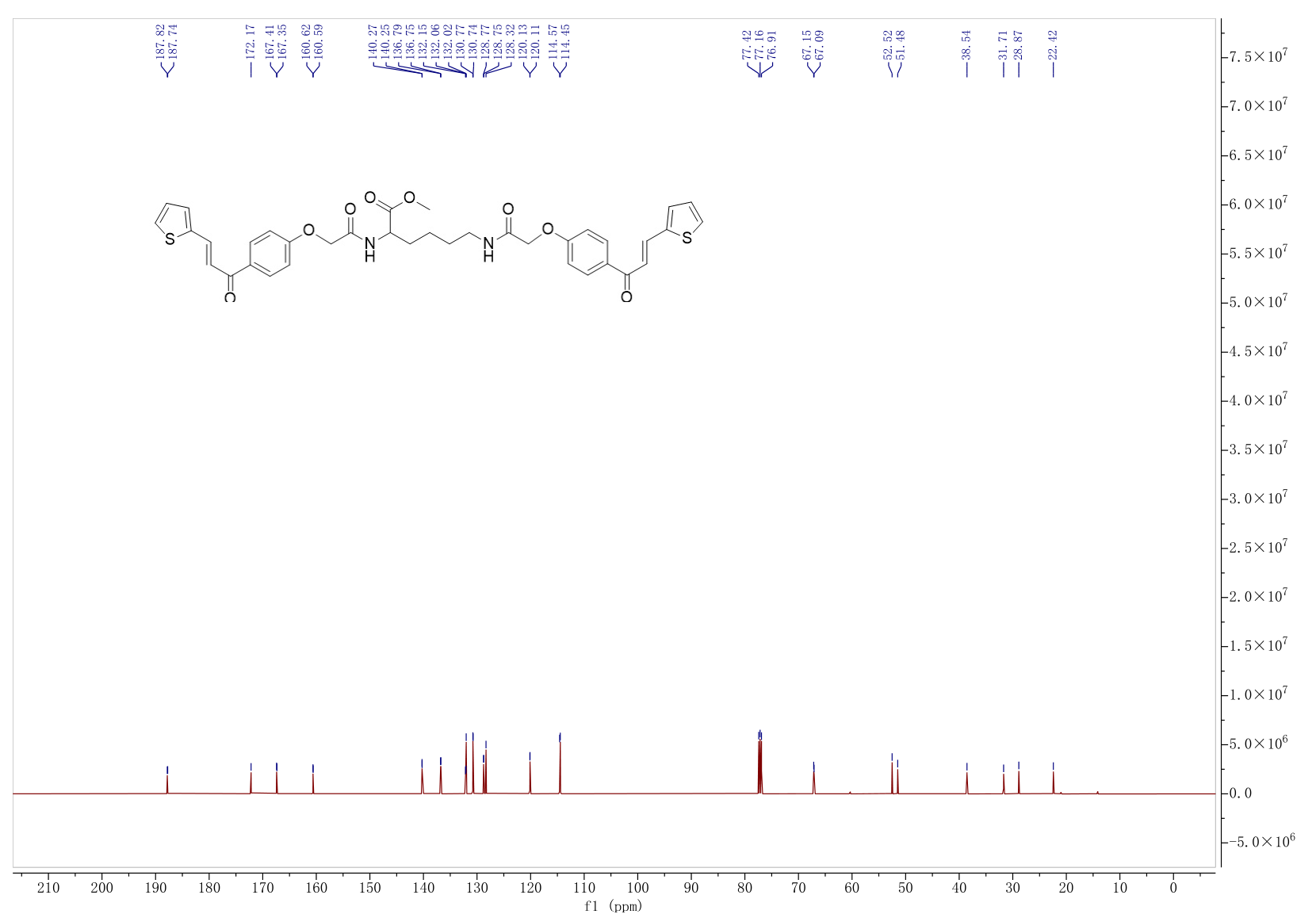


**Fig. S14.** ^13^C NMR spectrum of compound **3e** in Chloroform-*d*.


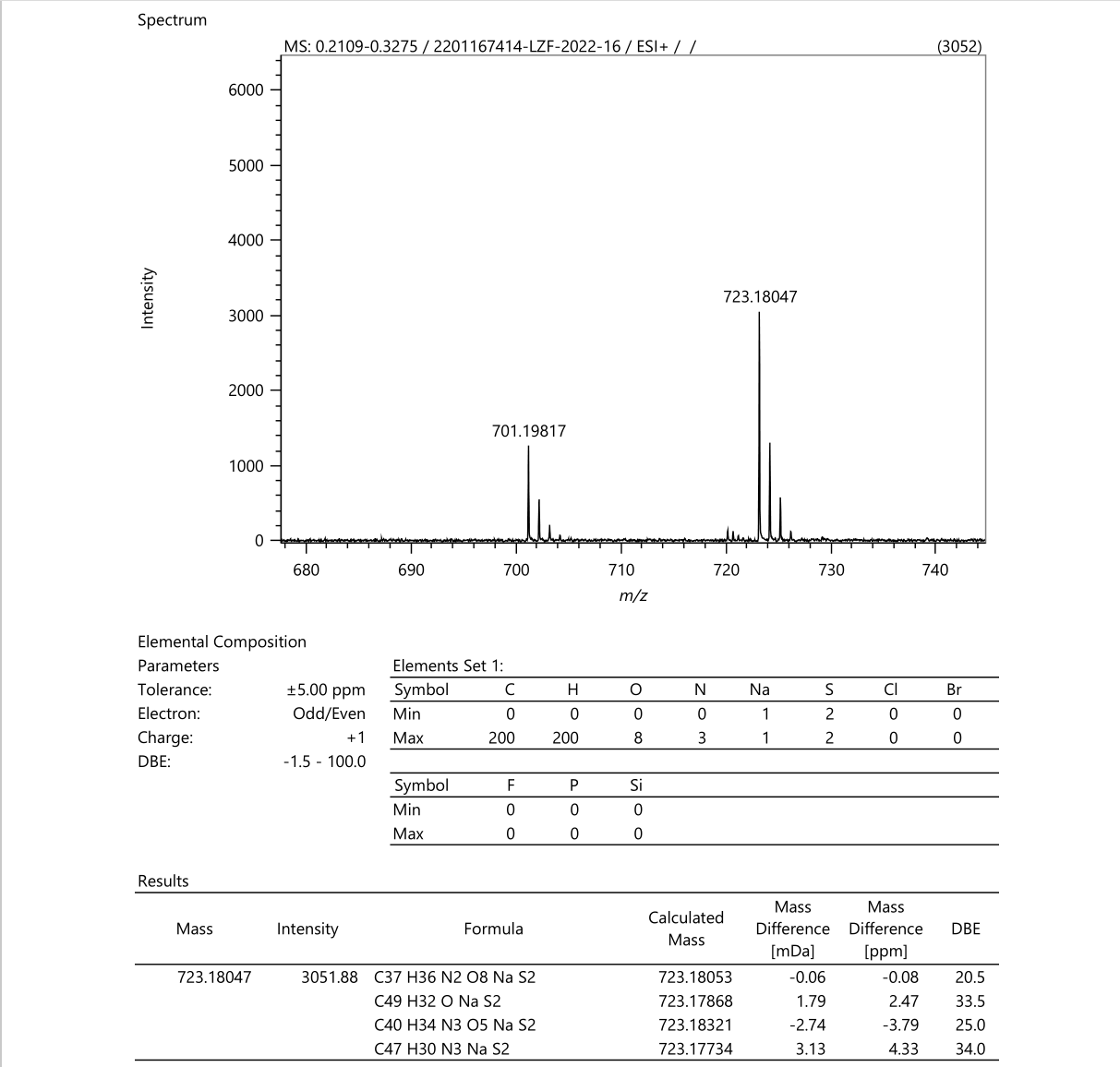


**Fig. S15.** HRMS of compound **3e**.


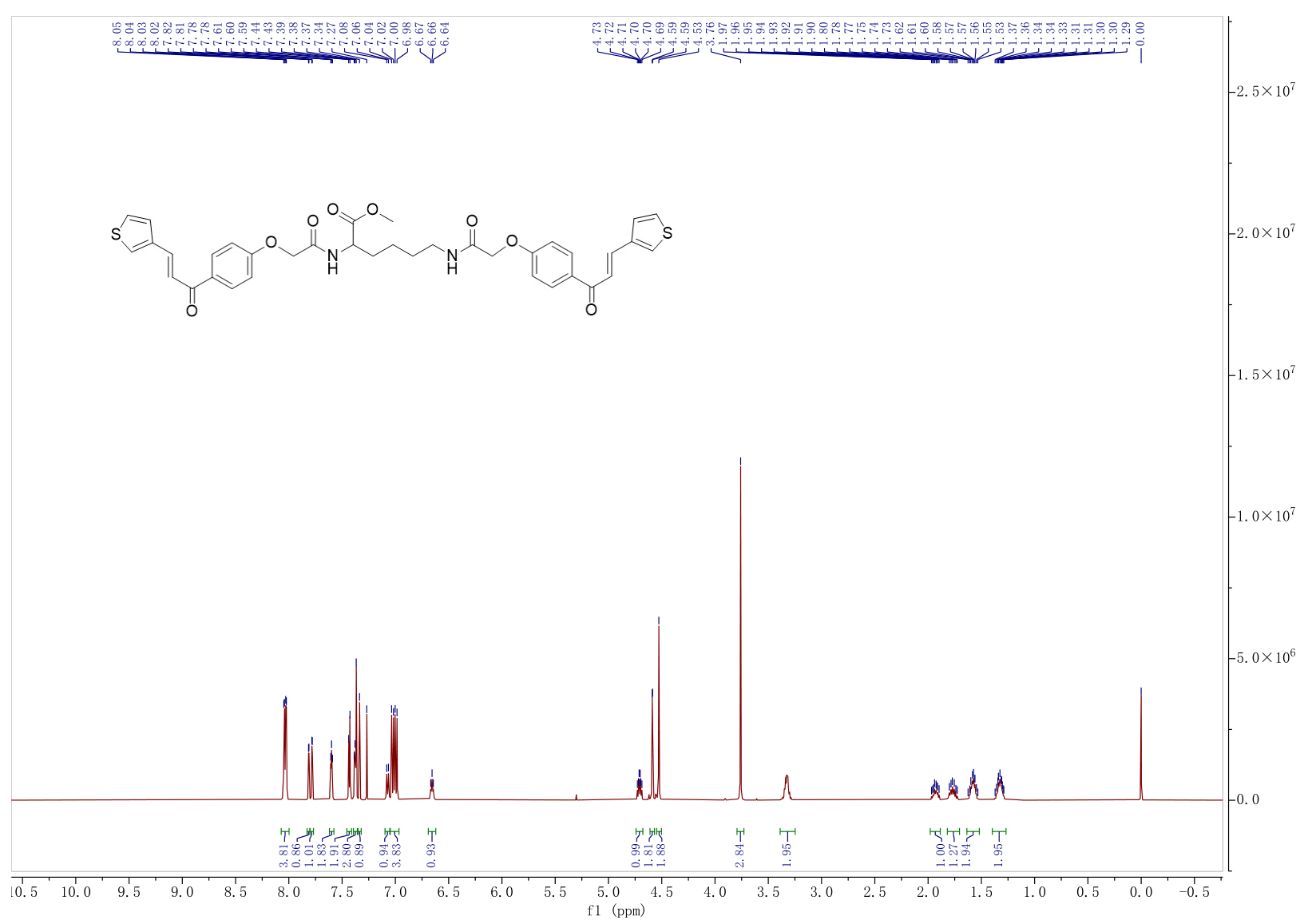


**Fig. S16.** ^1^H NMR spectrum of compound **3f** in Chloroform-*d*.


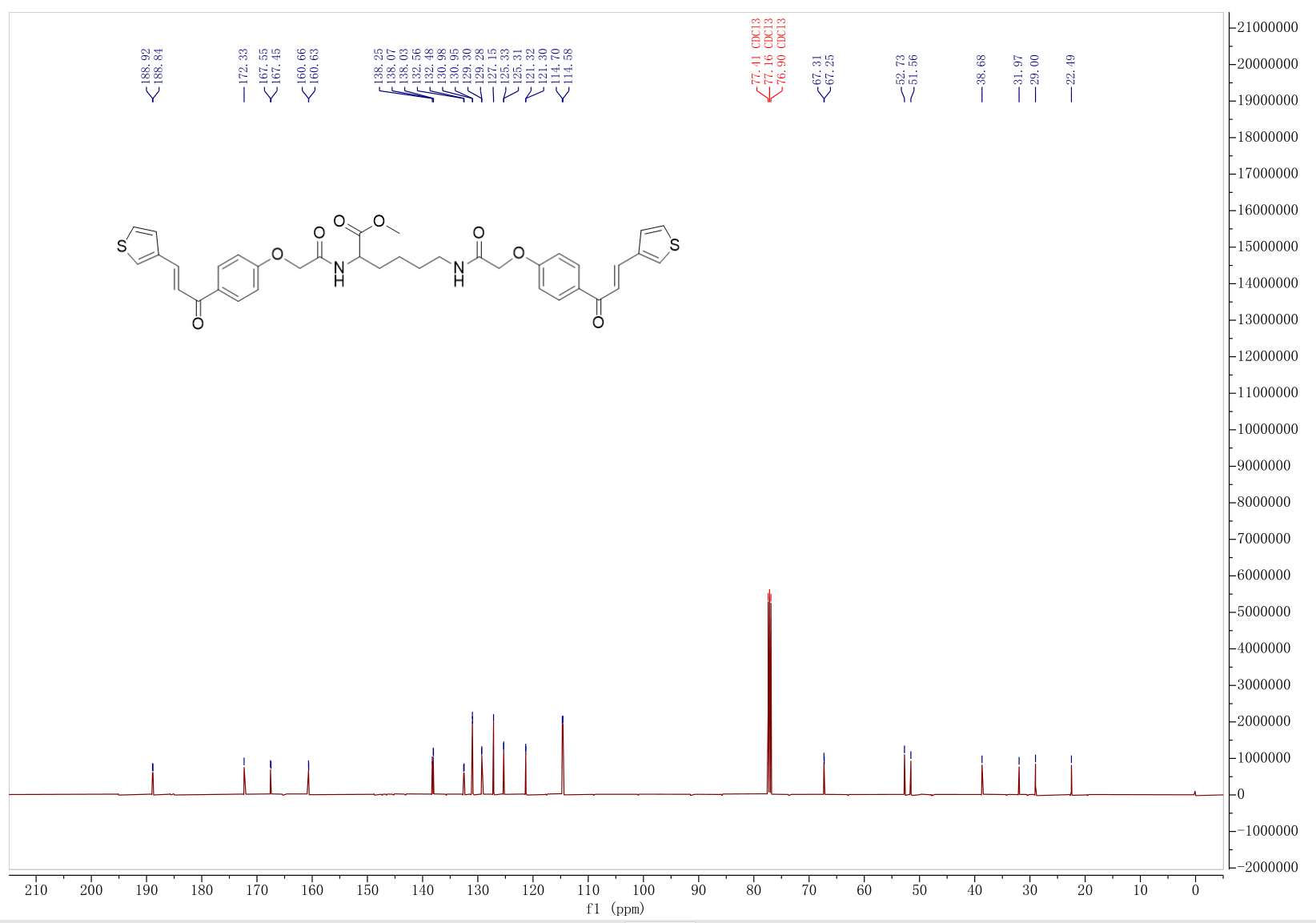


**Fig. S17.** ^13^C NMR spectrum of compound **3f** in Chloroform-*d*.


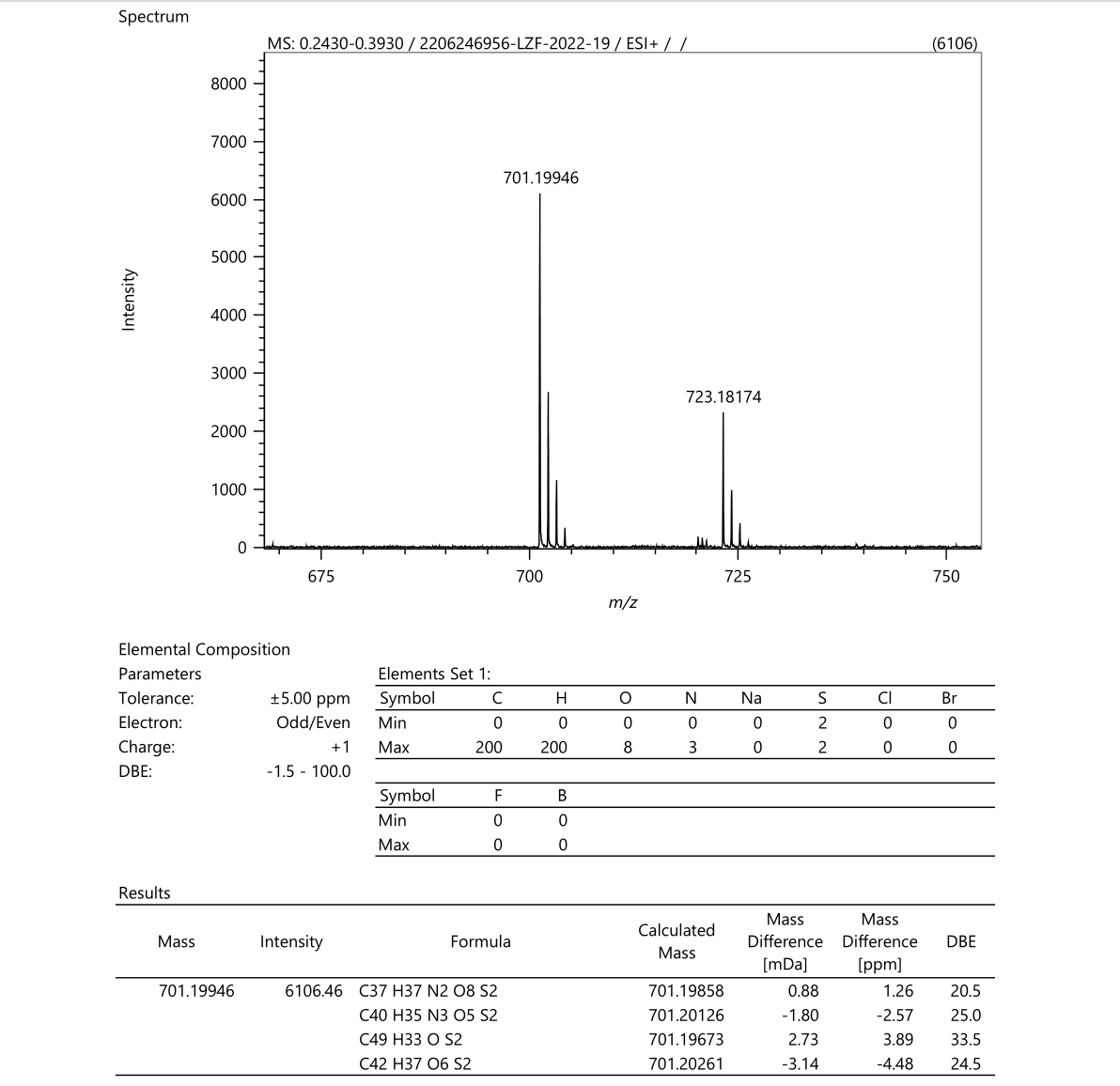


**Fig. S18.** HRMS of compound **3f**.


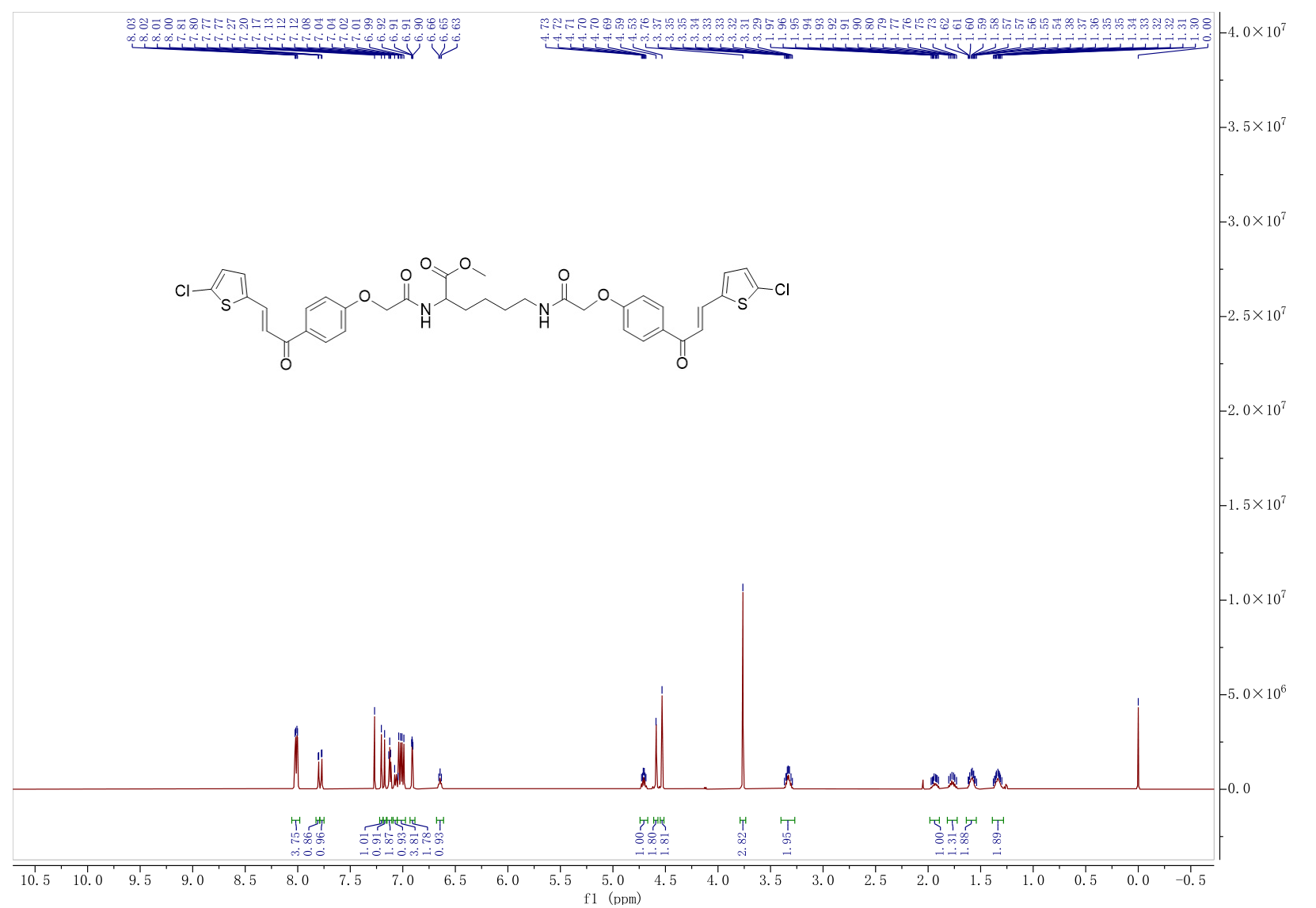


**Fig. S19.** ^1^H NMR spectrum of compound **3g** in Chloroform-*d*.


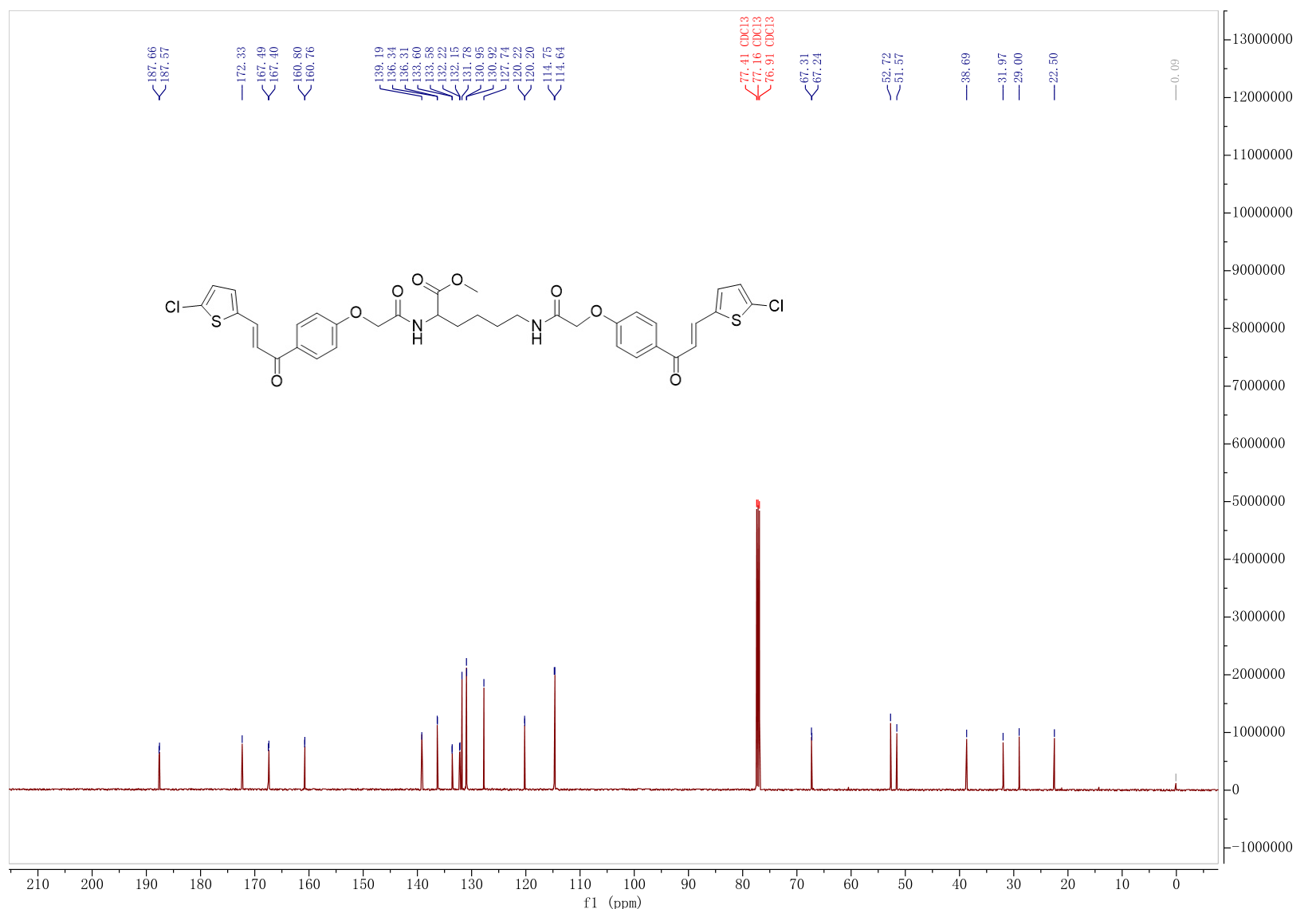


**Fig. S20.** ^13^C NMR spectrum of compound **3g** in Chloroform-*d*.


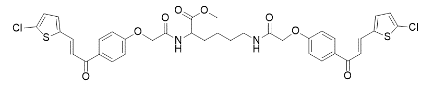

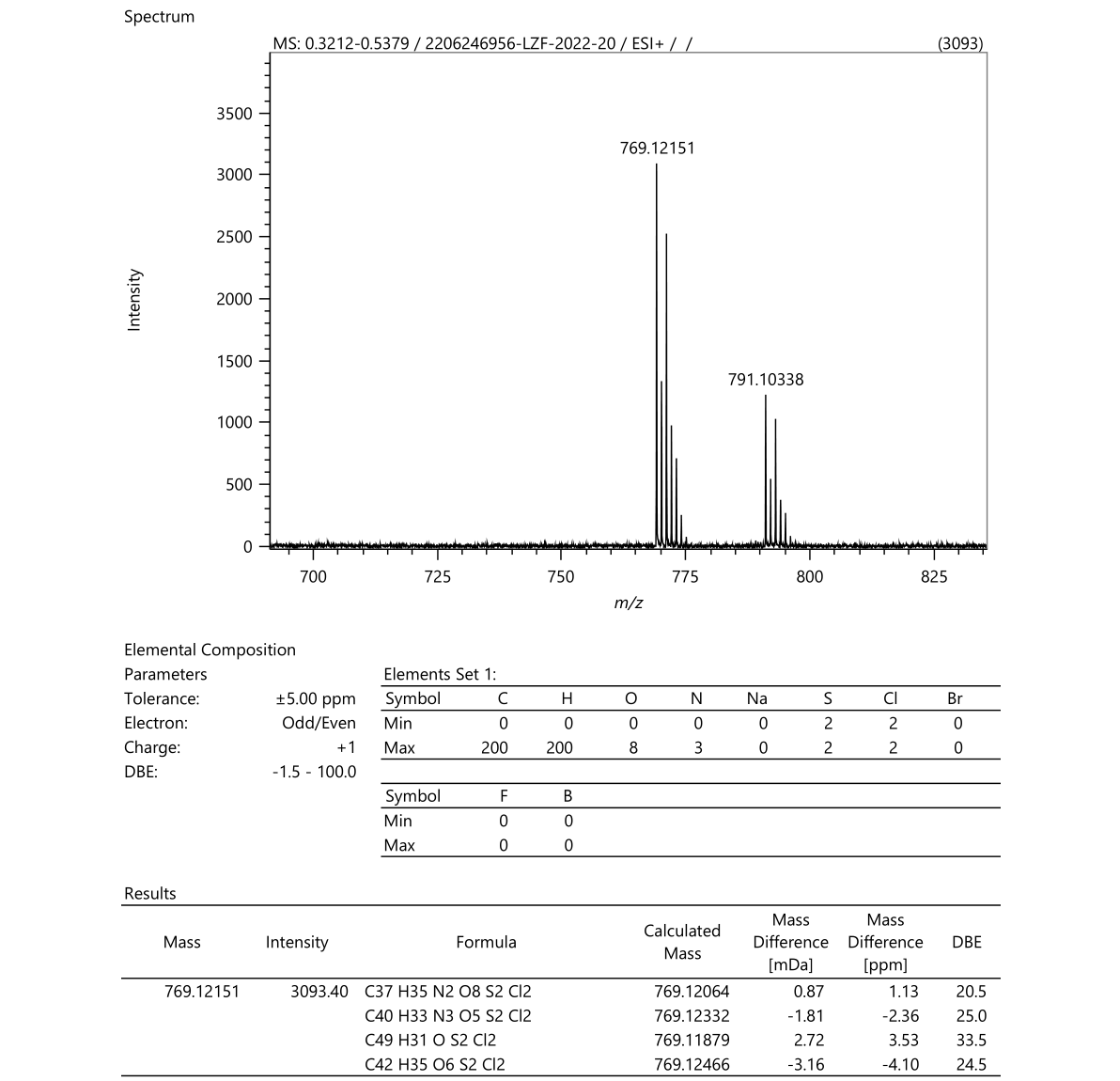


**Fig. S21.** HRMS of compound **3g**.


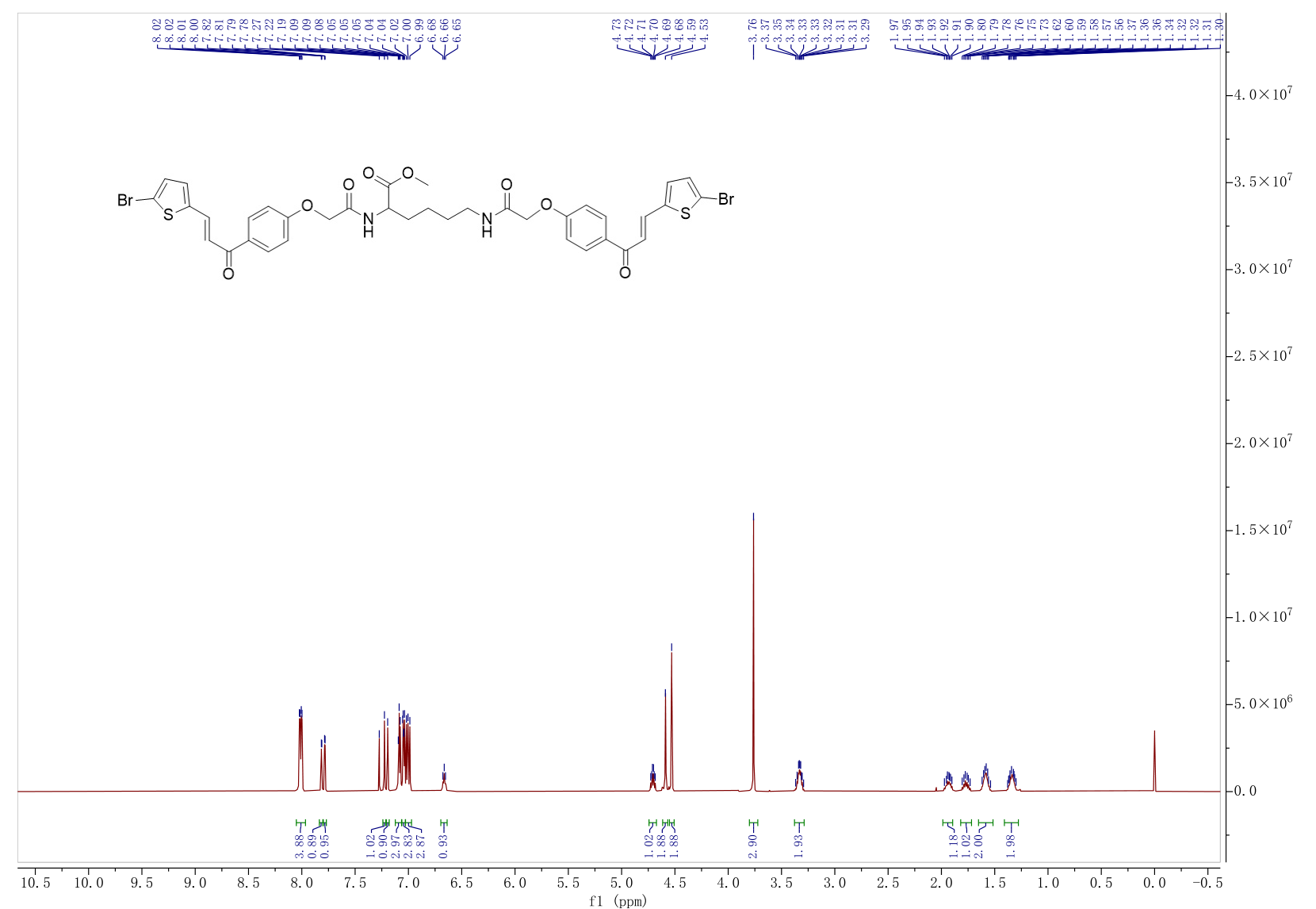


**Fig. S22.** ^1^H NMR spectrum of compound **3h** in Chloroform-*d*.


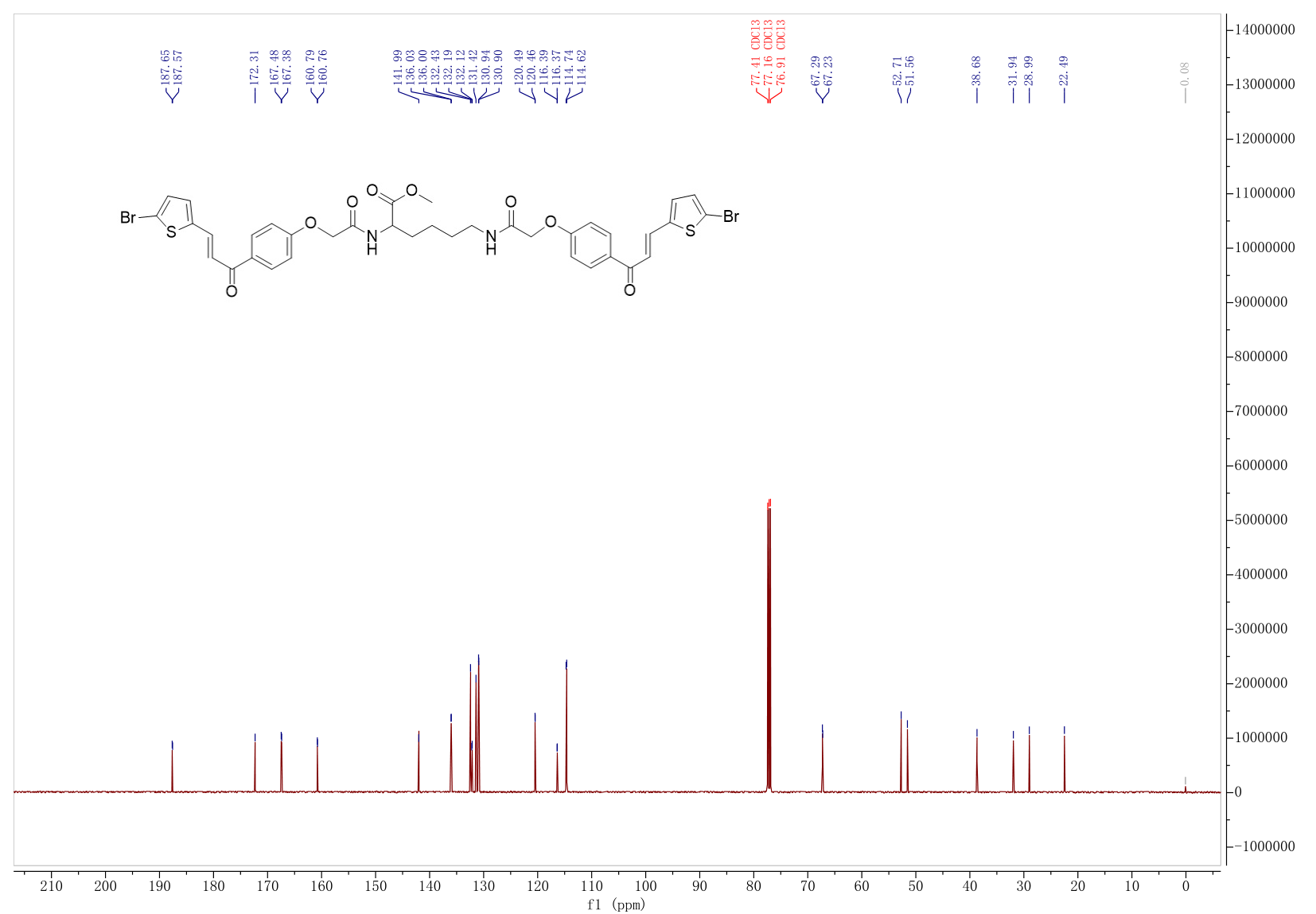


**Fig. S23.** ^13^C NMR spectrum of compound **3h** in Chloroform-*d*.


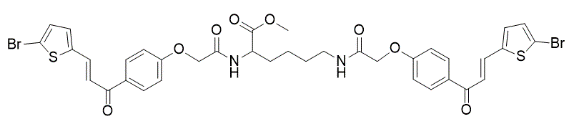

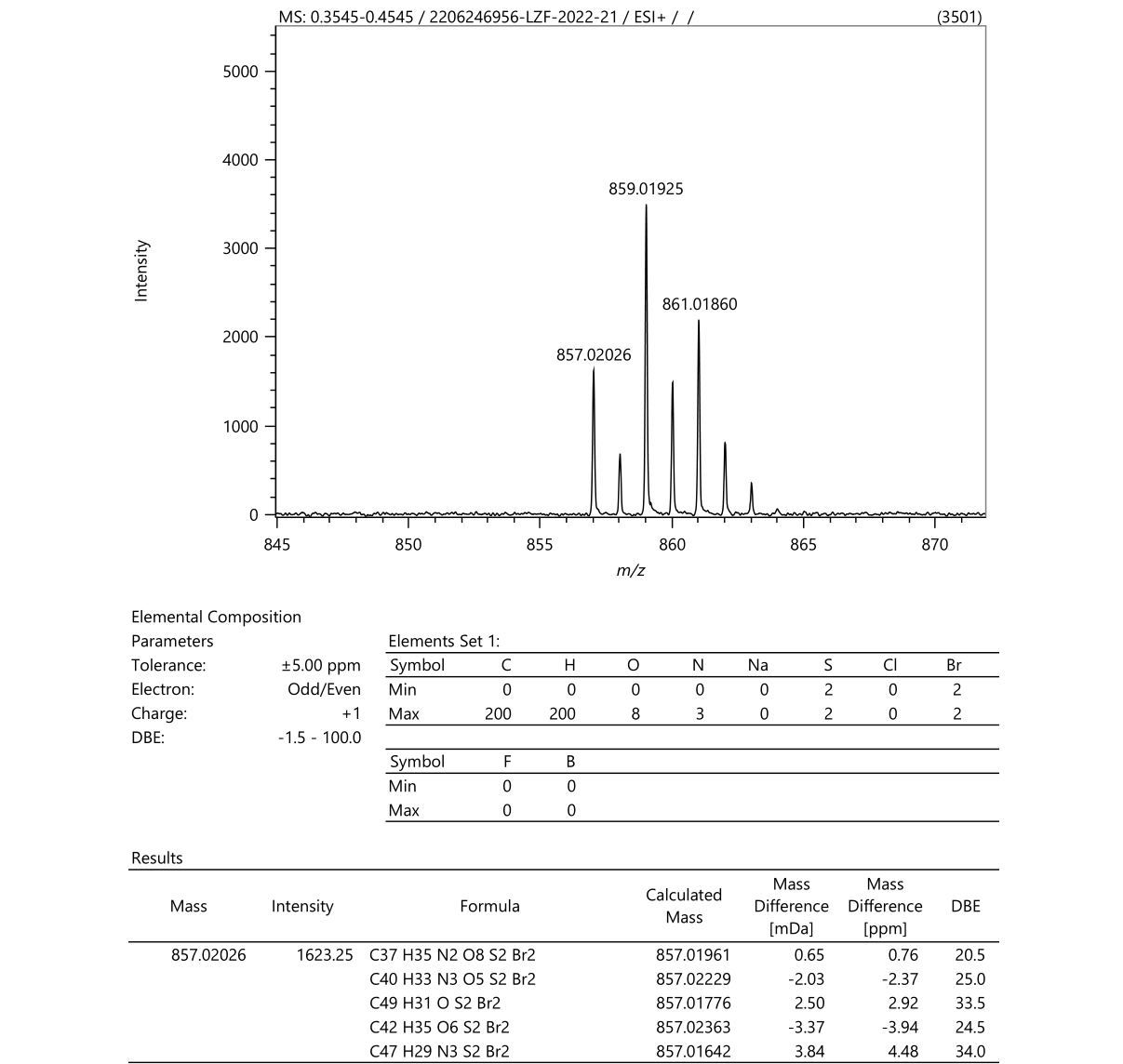


**Fig. S24.** HRMS of compound **3h**.


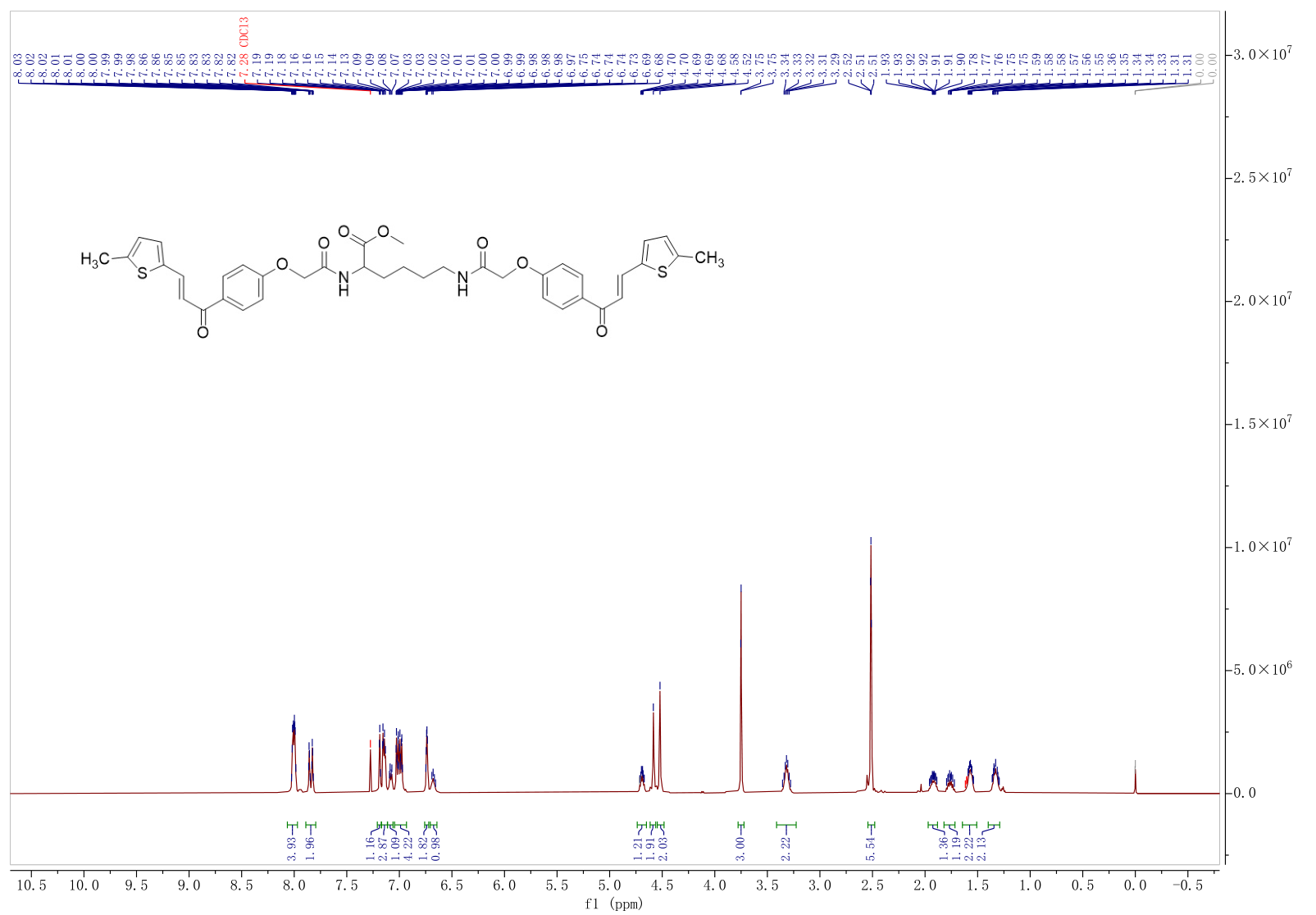


**Fig. S25.** ^1^H NMR spectrum of compound **3i** in Chloroform-*d*.


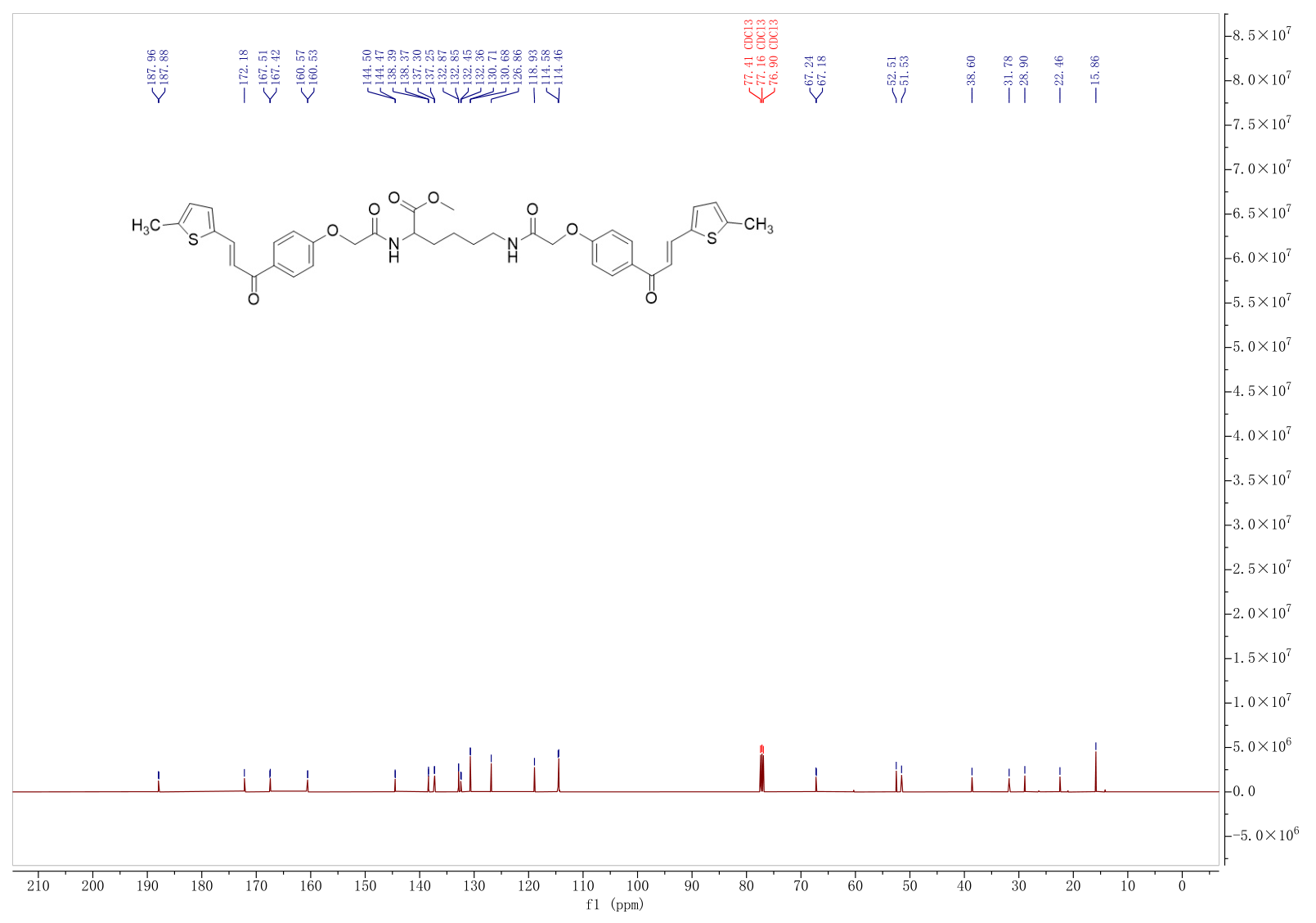
**Fig. S26.** ^13^C NMR spectrum of compound **3i** in Chloroform-*d*.


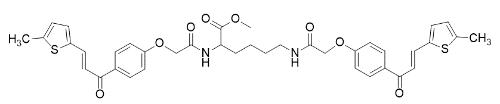

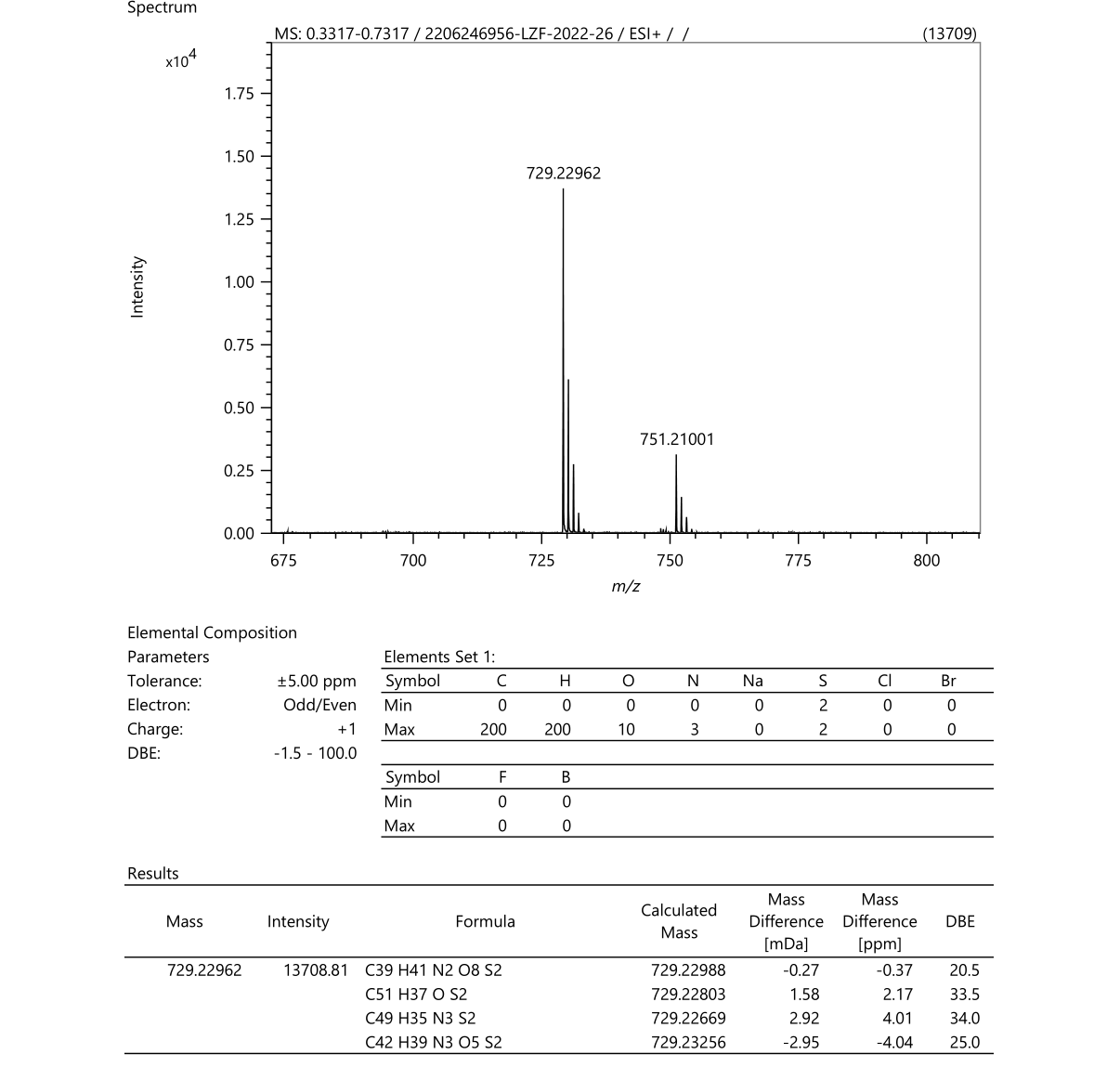
**Fig. S27.** HRMS of compound **3i**.


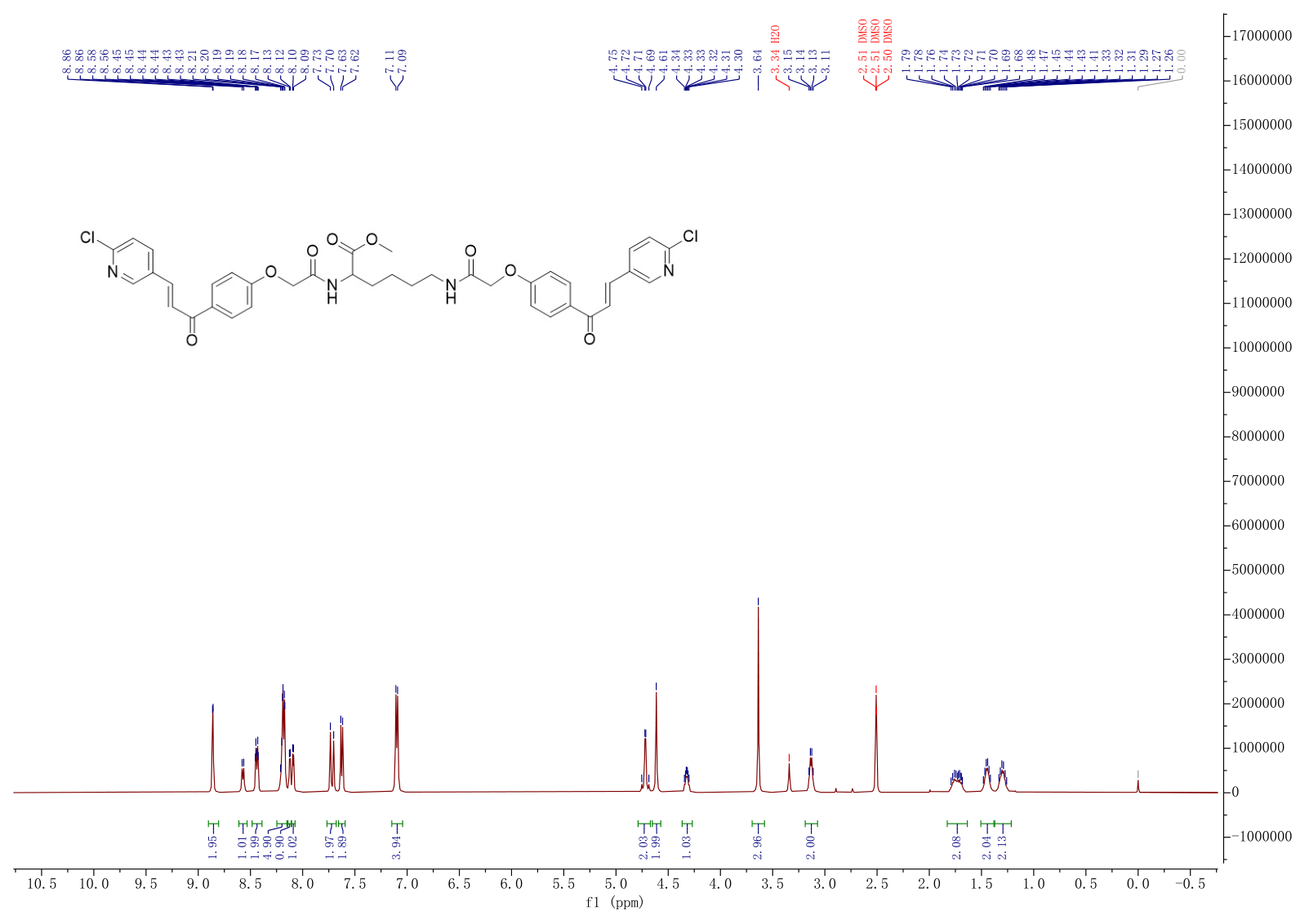


**Fig. S28.** ^1^H NMR spectrum of compound **3j** in DMSO-*d*_6_.


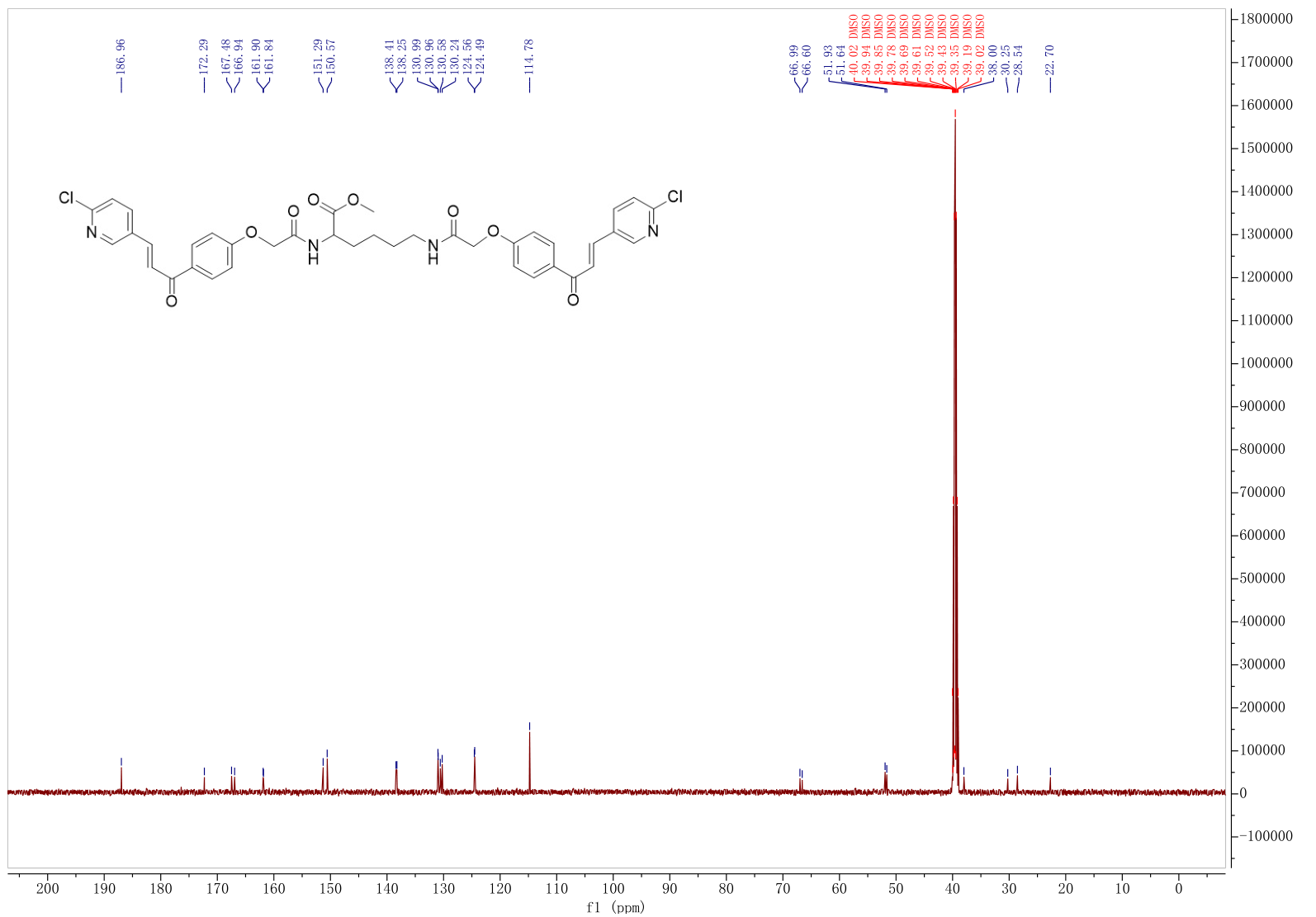


**Fig. S29.** ^13^C NMR spectrum of compound **3j** in DMSO-*d*_6_.


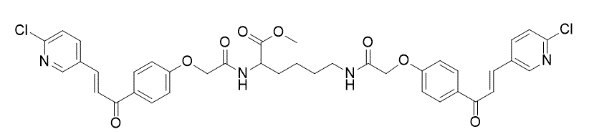

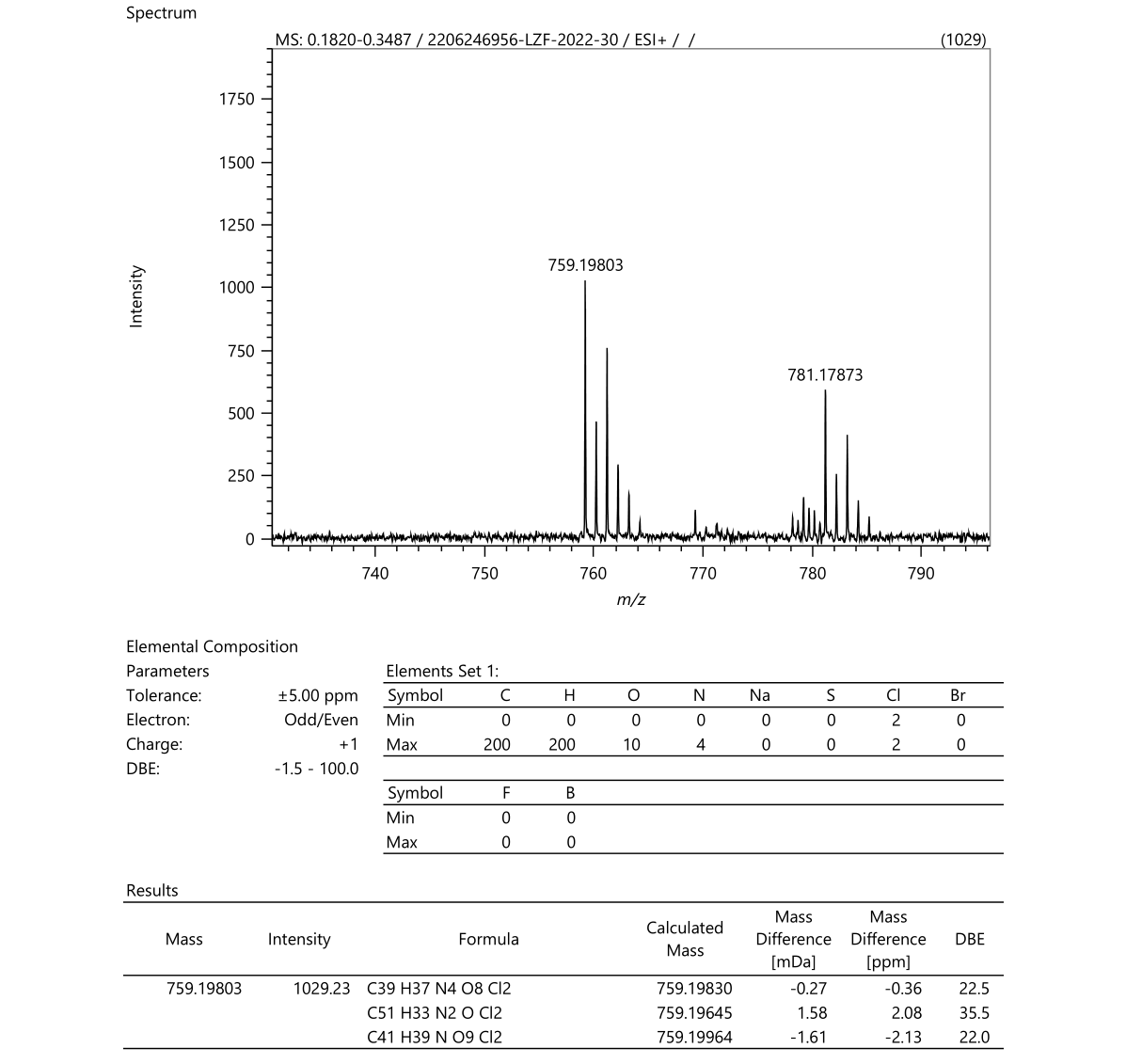


**Fig. S30.** HRMS of compound **3j**.


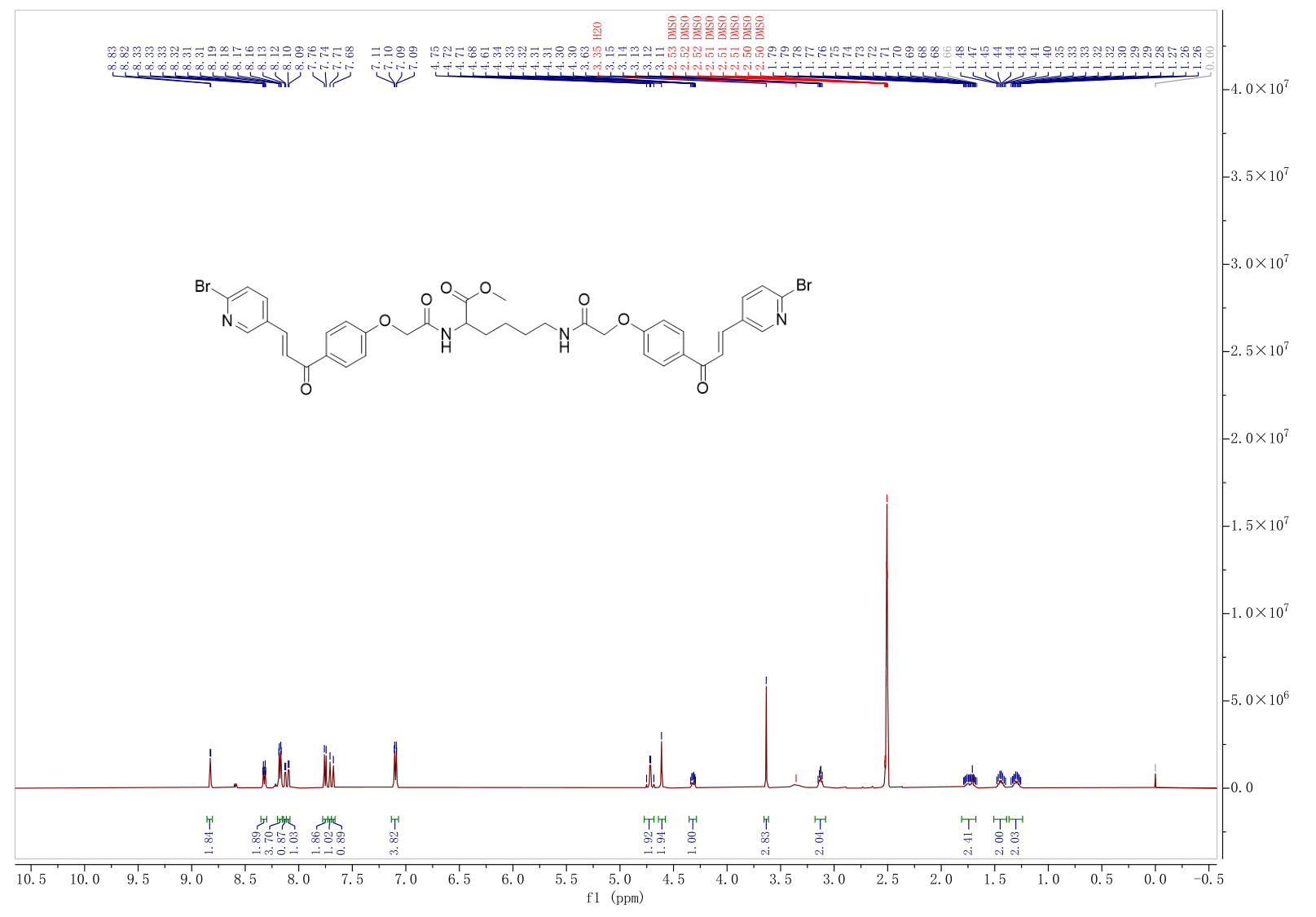


**Fig. S31.** ^1^H NMR spectrum of compound **3k** in DMSO-*d*_6_.


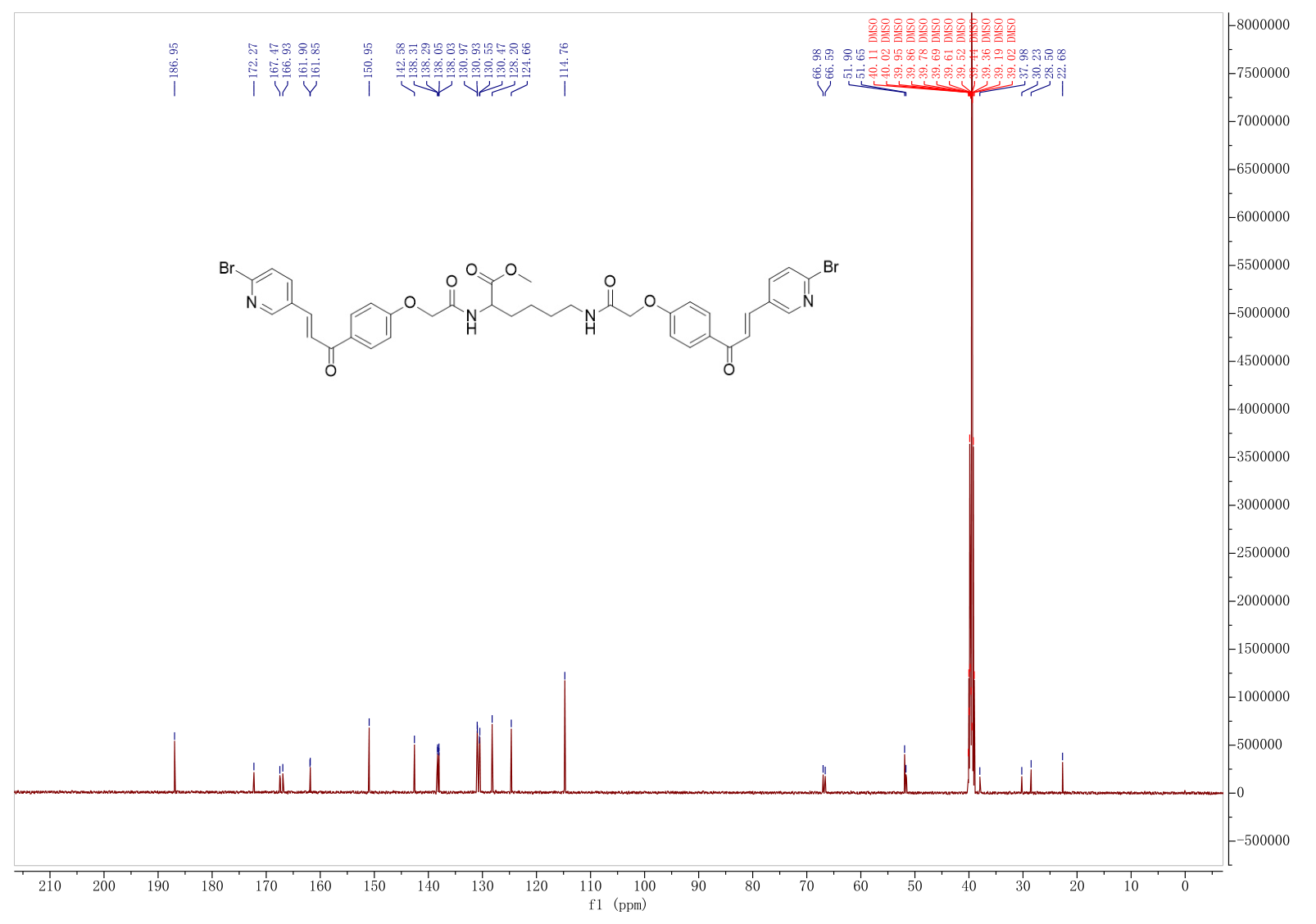
**Fig. S32.** ^13^C NMR spectrum of compound **3k** in DMSO-*d*_6_.


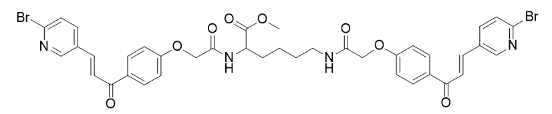

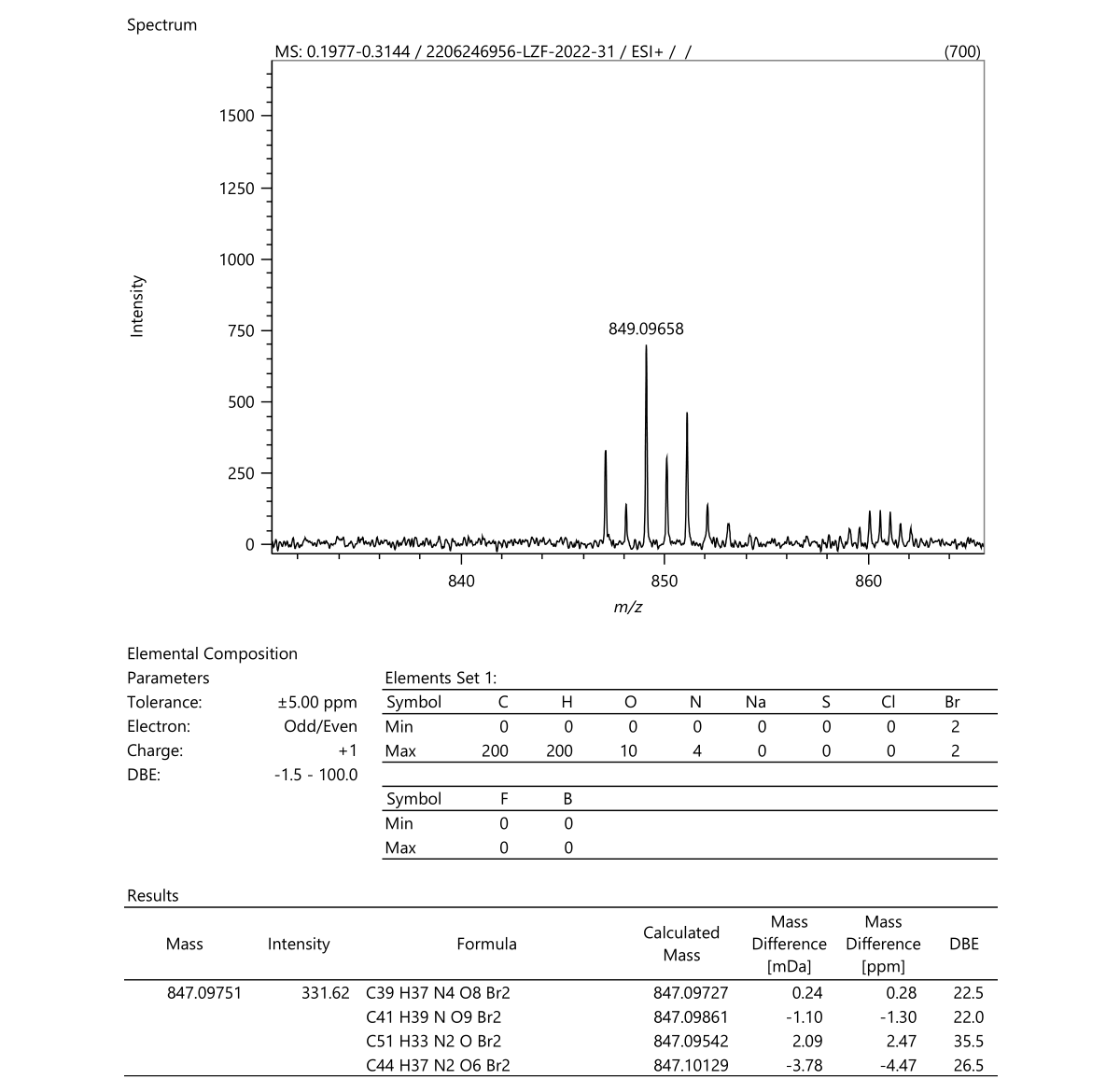


**Fig. S33.** HRMS of compound **3k**.


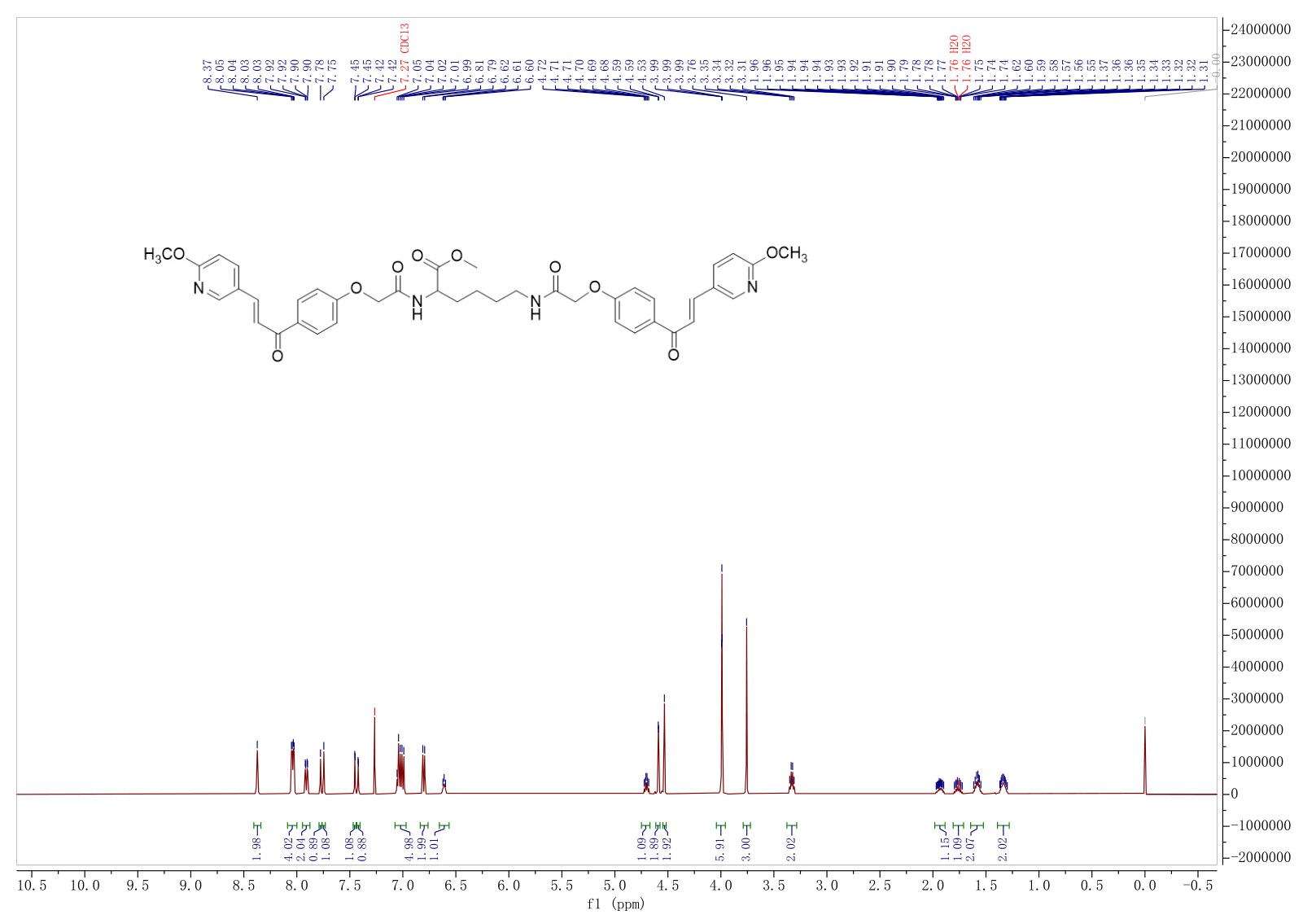
**Fig. S34.** ^1^H NMR spectrum of compound **3l** in Chloroform-*d*.


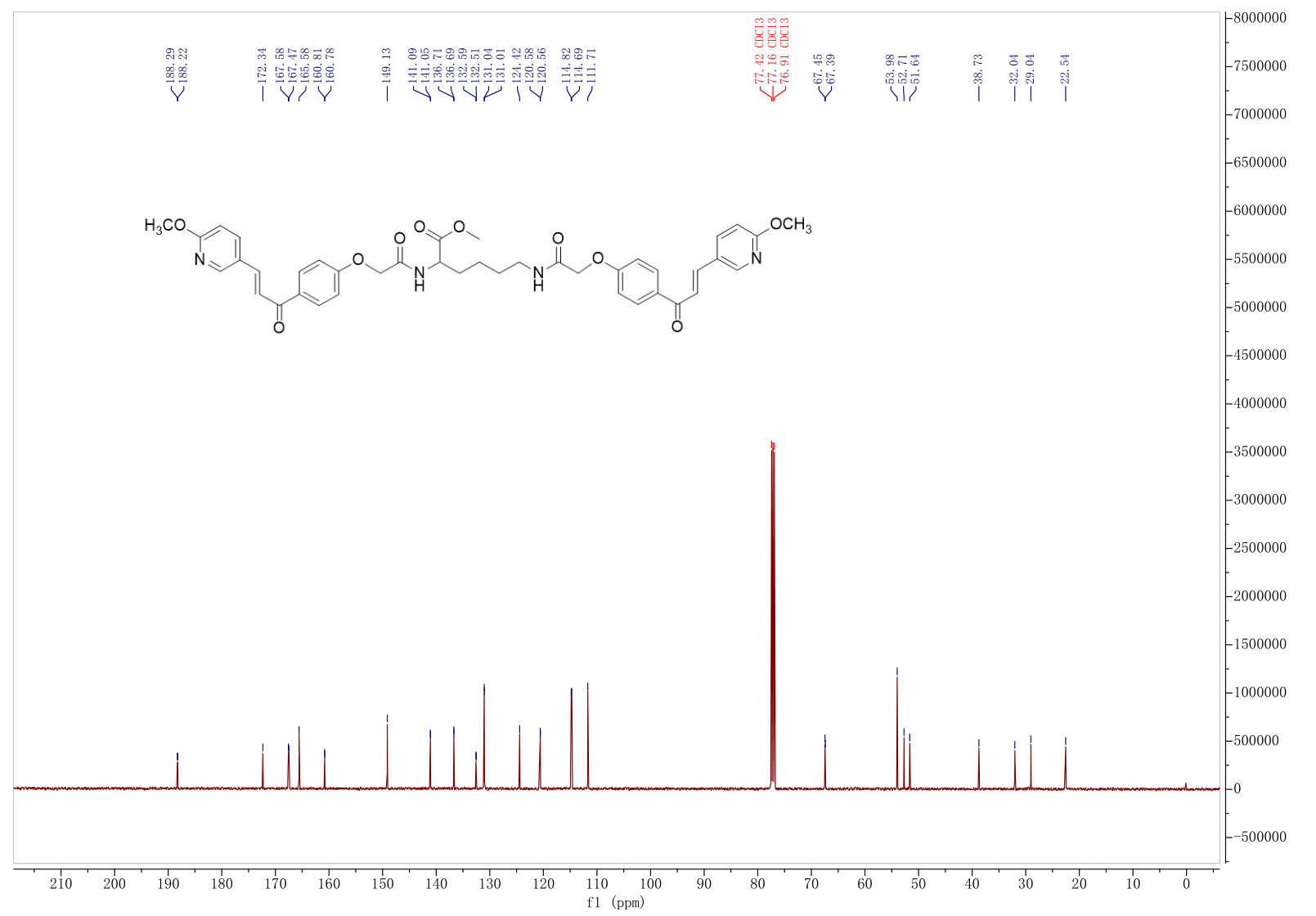


**Fig. S35.** ^13^C NMR spectrum of compound **3l** in Chloroform-*d*.


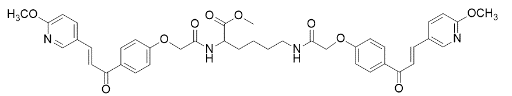

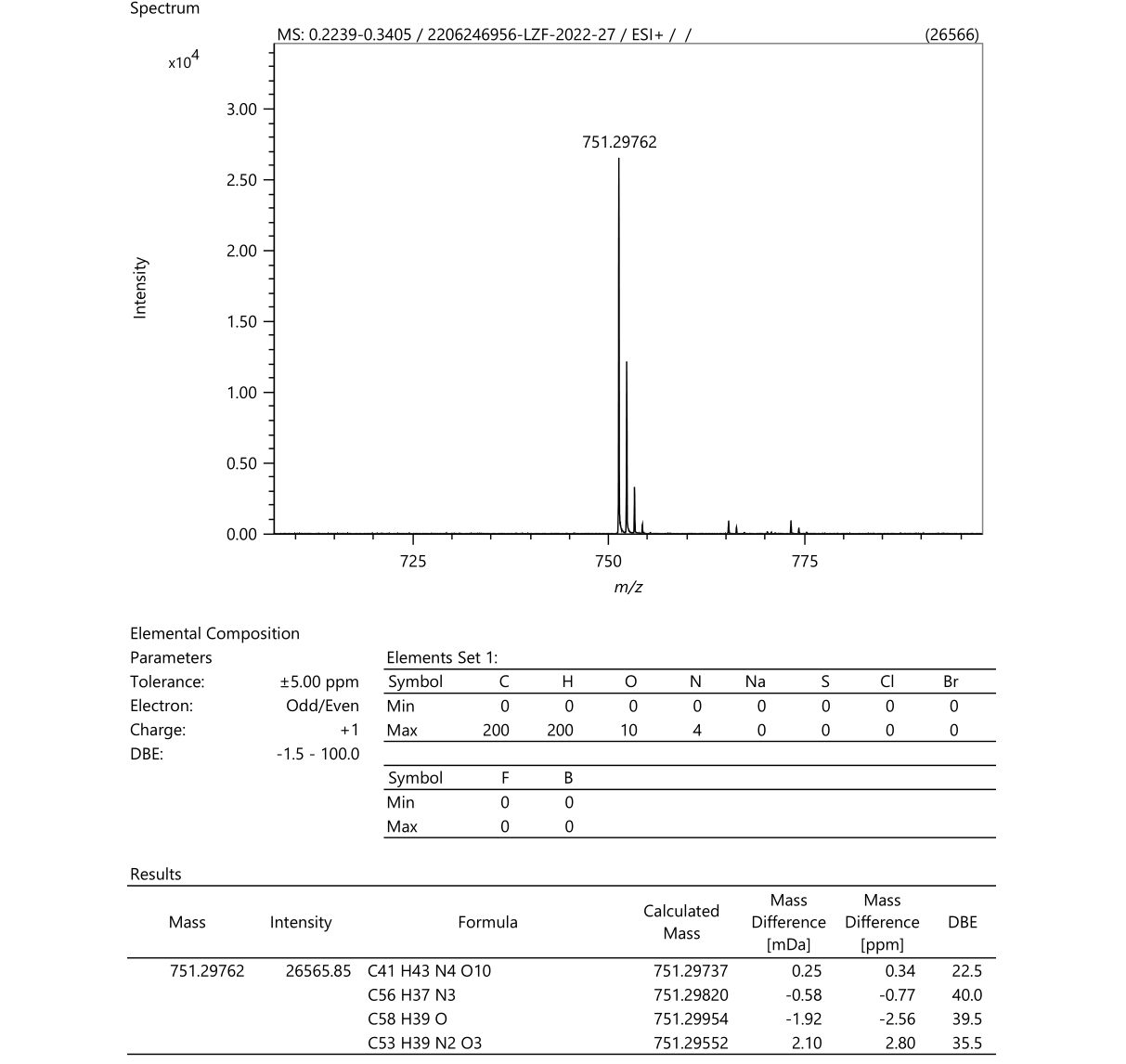


**Fig. S36.** HRMS of compound **3l**.
